# Gait and posturographic parameters of children with achondroplasia – a long-term pilot study

**DOI:** 10.1186/s12891-026-09780-3

**Published:** 2026-04-02

**Authors:** Mareike Hergenröther, Katja Palm, Klaus Mohnike, Kerstin Witte

**Affiliations:** 1 Sports Engineering / Movement Science Department, Faculty for Human Sciences, Otto-von- Guericke University, Magdeburg, Germany; 2 Children’ s Hospital, Otto-von-Guericke-University, Magdeburg, Germany

**Keywords:** Achondroplasia, 3DGA, Motion capture, Gait analysis, Children's development, Postural control

## Abstract

Walking and maintaining balance are essential for participating in daily life. For children with achondroplasia, reaching these milestones is with a delay and during growth, challenges persist for those skills. However, the impact of developmental changes on gait and balance in this population has not yet been investigated longitudinally. Therefore, this study aimed to examine the influence of growth on gait pattern and postural control in children with achondroplasia over a two-year period. 6 children with achondroplasia (whereas 5 are treated with vosoritide, a growth promoting peptide) and 24 children of average height participated in a 3D gait analysis and static bipedal posturographic (eyes open/closed and foam pad with eyes open/closed) measurements every six months. Using Nonparametric Analysis of Longitudinal Data and the smallest real differences, changes over time and between the cohorts were investigated. Results showed that the growth development between the cohorts were similar. Still, the key characteristics of achondroplasia were prominent and revealed significant differences for the anthropometric parameters. Spatio- temporal, kinematic parameters revealed additional significant differences for known characteristics like gait velocity, step- and stride length, cadence, step & stride time, stance- and swing phase and single support time, between the cohorts. Finally, posturographic parameters present significant differences in the foam pad eyes closed condition between the cohorts. Overall, all children show considerable variability for gait and posturographic parameters, with greater variability present children with achondroplasia. The findings of this work indicate that there are no general differences in the growth curves between children with achondroplasia and children of average height. However, the different baseline of both cohorts, with the vosoritide treatment and the already changed gait pattern of achondroplasia must be considered as additional factors. Further research is needed to investigate broader age groups, possible gender differences and greater sample sizes, e.g. through a multi-center study.

**Supplementary Information:**

The online version contains supplementary material available at 10.1186/s12891-026-09780-3.

## Introduction

 The ability to walk and maintain balance is fundamental for participation in activities of daily living and for independent living. In children, these skills begin to develop in early infancy and may continue to mature into adolescence, with full development achieved later in life [1, 2]. Neurological and orthopedic conditions, such as cerebral palsy or joint malalignments, are known to impair gait patterns [3] and balance control [4]. In individuals with achondroplasia (ACH), similar challenges have been reported in cross-sectional studies [5, 6].

ACH is a form of disproportionate short stature, that occurs in approximately 1 in 25,000–30,000 live births per year worldwide [7]. Due to a mutation in the FGFR3 growth hormone people with ACH have a decreased growth of extremities, while sitting height remains within the lower normal range [8], causing disproportion in the arms and legs which often leads to a genu varum and/or valgum of the legs, with genu varum being more prominent [8, 9]. Individuals with ACH typically present with musculoskeletal features including lumbar hyperlordosis, thoracic hyperkyphosis, and inherent spinal stenosis, which may result in pain, paraesthesia, and numbness in the lower extremities [7]. Reduced foot length is also commonly observed in this population [10]. Children with ACH frequently demonstrate delays in gross motor milestones, such as standing and walking. These limitations may persist into adulthood, where activities of daily living, including prolonged walking and stair negotiation, remain challenging [7].

Previous studies regarding the gait of people with ACH have shown that during walking an increased anterior pelvic tilt and an increased flexion pattern of the hip, knee, and ankle, as well as increased knee valgus and/or varus and hip abduction are present [6, 10–13]. Partly those characteristics can be related to the ligament laxity and hypermobility of people with ACH [7] as well as the shorter leg length. Regarding the time-distance parameters, a shorter step- and stride length, a decreased walking speed as well as an increased cadence is stated. Differences are still present after normalizing the parameters via the leg length [6, 12, 14, 15]. However, the evidence base on gait patterns in individuals with ACH is sparse and fragmented, with considerable discrepancies reported for normalised parameters.

Regarding the postural control of people with ACH, limited information is available. Three studies investigated the postural stability of people with ACH using X-ray images [16–18]. Cai et al. [17] could show that an imbalanced spine was present for 13 of 28 patients, with a tendency for imbalance in a later age span. Hong et al. [18] could show significant differences for multiple spine parameters for people with ACH compared to a control cohort of adults of average height and no spinal deformities and Karikari et al. [16] could confirm this for young children with ACH. Alves et al. [19] and previous work of this authors group investigated cross-sectional the postural control of people with of ACH during four different postural control tasks. Regarding the previous work of this authors’ group, no significant differences were found. However, some parameters demonstrate large effect sizes, suggesting potential differences between children with ACH and children of average height (CAH) [5], although these findings should be interpreted with caution given the limited evidence base. Alves et al. [19] compared individuals with ACH who had undergone leg-lengthening surgery with those of natural height. The leg-lengthened group showed higher values in linear postural control measures, including centre of pressure (CoP) displacement, total excursion, amplitude, and ellipse area, whereas the natural-height group exhibited higher values in nonlinear metrics, such as sample entropy [19], While these findings have been interpreted as reflecting more variable and adaptable postural control in the natural-height group, the distinction between adaptive variability and instability remains unclear. Furthermore, the reported positive association between physical activity—particularly muscle strength—and postural stability was preliminary, and causality cannot be inferred [19]. Knowing the above-mentioned changes in the gait and postural control of people with ACH, there is still no study investigating children with ACH over time and therefore seeing the development of the anthropometric as well as the motoric skills regarding their gait and postural control. Those are beneficials key aspects in the general development of children, especially for children with ACH Understanding in a deeper way the differences and challenges for this cohort, can provide valuable information regarding therapeutic treatments during infancy, later childhood and adolescence.

With the use of modern technologies like motion capture systems and force plates it is possible to fulfill three-dimensional gait analysis (3DGA) and postural control analysis to observe desired parameters of the gait pattern and postural control. 3DGA provides the opportunity to analyze the movement pattern of a person as a non-invasive method [20]. Retroreflective markers will be mounted with skin-friendly tape to the skin of a person and allow unrestricted movement during an analysis. Via synchronization, force plates and motion capture systems can be used simultaneously and provide the opportunity for more output parameters [20].

Therefore, the aim of this study is to longitudinally investigate gait patterns and postural control in children with ACH compared with a control cohort of CAH over a 2-year period. In addition, this study seeks to establish a methodological framework and prospective statistical approach to inform future research with larger sample sizes.

## Methods

A comparative longitudinal pilot study was conducted over a 2-year period, with assessments performed at 6-month intervals. A total of 24 CAH and 16 children with ACH, aged 7–13 years, were initially enrolled. However, only six children with ACH completed all four assessment time points and were included in the final analysis (see Table 1), resulting in a small final sample size that limits statistical power and generalisability. Dropout was primarily attributed to low motivation and the time demands associated with gait laboratory testing (approximately 60 min per session). The final analysis therefore included only participants with complete datasets, alongside the corresponding CAH cohort.

All participants were required to ambulate independently without assistive devices for a minimum distance of 10 m. Exclusion criteria included any lower extremity injury within the preceding 6 months. Additionally, children in the CAH group were required to have no clinically relevant malalignment of the knee or ankle joint axes. Participants in the CAH group were recruited through local schools and sports clubs.

Children with ACH were invited through the children’s university hospital, when they were visiting for their routine medical checkup. Five out of the six children with ACH are treated with Vosoritide, a growth promoting peptide (see Table 7). In a current study 186 children with achondroplasia treated for 3 years with vosoritide an additional 5.6 cm on average was achieved [21]. Similarly, a recent meta-analysis reported an increase in annualized growth velocity of 1.91 cm/year (95% CI: 1.53–2.29, *n* = 121) [22], resulting in a growth rate analog to healthy children. Sawamura et al. [23] could prove, that the spine and lower limbs tend to shift slightly to the normal range, after a year of treatment. However, long term results and benefits of this treatment are still unknown. Before participation, children and their legal guardians were orally briefed on the study procedures, and all questions were addressed. Written informed consent was obtained from both the participants and their legal guardians. This study was approved by the Ethics Board of Otto-von-Guericke University, Magdeburg, Germany (Approval No. 82/23) and was conducted in accordance with the Declaration of Helsinki. Participant recruitment occurred between January 2023 and November 2025. In Table 6 additonally the weekly physical activities of all children with ACH is listed.

**Table 1:** Description of the anthropometric parameters for children with ACH and CAH at each time point. Presented as median and IQR [1–3]. ACH = Achondroplasia. CAH = Children of average height. IQR = Interquartile ranges, T1 = Timepoint 1, T2 = Timepoint 2, T3 = Timepoint 3, T4 = Timepoint 4

	ACH								
	T1		T2			T3		T4	
Parameter	Median	IQR [1–3]	Median	IQR [1–3]		Median	IQR [1–3]	Median	IQR [1–3]
Height (cm)	110.7	105.98–115.13	113.55	108.78–118.63		117.8	113.66–121.35	120	116.23–123.45
Weight (kg)	28.65	25.30–31.40	31.16	27.38–34.71		33.5	29.33–36.13	35.7	33.54–38.39
Age (years)	10.43	9.03–10.80	11.2	9.70–11.35		11.69	10.20–11.83	12.32	11.37–13.16
Sitting Height (cm)	72.5	68.73–75.30	74.8	72.25–78.40		77.2	72.73–80.48	78.9	77.98–80.95
Leg Length (cm)	43.1	41.48–46.13	45.05	42.01–47.83		46.15	43.76–48.76	48.18	45.40 -49.53
Standing Sitting Ratio (%)	65.5	64.25–66.75	66.5	66–67		66	65.25–66.00	67	67
Foot Leg Ratio (%)	42	41–43.75	42.5	41.25–43.75		43	42.25–45.25	43	42.25–43.75
Pelvic Tilt (deg)	22.45	18.08–26.08	21.28	19.14–24.69		20.48	18.03–23.28	26,7	24.27–33.56

	CAH								
	T1		T2			T3		T4	
Parameter	Median	IQR [1–3]	Median	IQR [1–3]		Median	IQR [1–3]	Median	IQR [1–3]
Height (cm)	139.8	123.50–146.3	140.6	127.10–145.50		145.05	130.23 -150.33	148	134.38–154.88
Weight (kg)	29.65	24.80–33.25	32.46	26.30–34.47		33.81	30.17–37.08	37.38	30.65–40.60
Age (years)	8.57	7.33 − 10.49	9.07	7.86–10.96		9.53	8.36–11.44	10.04	8.86–11.96
Sitting Height (cm)	73.25	67.63–76.13	73.5	67.88–75.75		75.45	69.70–77.35	75.35	71.88–79.85
Leg Length (cm)	73.38	64.22–76.16	74.1	65.18–78.48		76.23	67.33–80.13	78.18	69.18–83.00
Standing Sitting Ratio (%)	54	52.00–55.00	52	51.00–53.00		52	51.00–54.00	52	51.00–54.00
Foot Leg Ratio (%)	31	30.00–32.00	32	30.00–33.00		31	30.00–32.00	31	30.00–32.00
Pelvic Tilt (deg)	15.16	12.77–17.60	13.36	8.87–16.93		13.5	10.25–16.81	15.86	10.92–18.90

### Procedures

Data collection took place at the movement laboratory of the authors’ university, utilizing a 13 infra-red-light Vicon camera system (Vicon Vue Cameras (200 Hz), Vicon Motion Sytem, United Kingdom) and two in the ground integrated force plates (1000 Hz) (AMTI MiniAMP MSA- 6, AMTI, Watertown, USA). Both were run via the Vicon Nexus software and therefore synchronized for the recording of the trials.

All participants were asked to wear minimal skintight clothes and to walk barefoot. Prior to the data collection, participants were asked about their well-being and been orally instructed about the task. After the measurement of specific anthropometric data, 28 retroreflective markers were placed on specific anatomical landmarks following a modified plug-in gait model [24]. A static calibration trial was recorded to develop an individualized model for each participant.

### Posturographic measurements

Participants were instructed verbally about the task including the following four conditions: flat surface and eyes open (EO), flat surface and eyes closed (EC), foam pad and eyes open (FEO), and foam pad and eyes closed (FEC). Each condition was performed for 30 s with the participant being barefoot. For each condition, the participant stood with a pelvic width stance on a force plate and had the arms in a rigid extended position next to the body. Pelvic width was calculated as the distance between the anterior iliac spine markers. Participants were instructed to look at a fixation point approximately 8 m in distance without any movement. For the conditions with the foam pad (AIREX^®^), the force plate was leveled to zero again with the foam pad lying on it. Trials were recorded through Vicon Nexus 2.15–2.18.1. Each trial was automatically stopped with a time range set in the Vicon System after 30 s. In addition, the pelvic tilt was measured through the used mod. PiG. Model (see Table 1). The pelvic tilt provides a greater understanding about the static and dynamic postural control [25] and therefore was chosen as an additional parameter to provide a better understanding of the postural control of children with ACH and the impact of their anterior shifted pelvic tilt, due to hyperlordosis, to their postural control.

### Gait analysis

Participants were instructed to walk multiple times along an approximately 10-meter walking path at their self-selected walking speed. The trials were recorded using the Vicon Nexus software.

### Data processing

#### Gait parameters

Trials have been post-processed via Vicon Nexus and exported via Vicon Polygon to obtain data samples defined as 52 data points per gait cycle. Six trials were chosen for each participant, due to the difficulty of measuring decent trails for children with ACH. Spatio temporal parameters were exported via Vicon Quick reports. Spatio-temporal parameters were normalized by leg length, following the protocol of Hof [28]. The median was used after confirming symmetry between the left and right legs for everyone. The spatio-temporal parameters of interest included walking speed, stride length, step length, single- and double stance time, stance- and swing phase and cadence, as well as the normalized versions of walking speed, cadence, stride- and step length. Kinematic parameters of interest included pelvic tilt, pelvic obliquity, pelvic rotation, hip flexion/extension, hip adduction/abduction, hip rotation, knee flexion/extension, ankle dorsiflexion/plantarflexion, foot progression angle based on the selected parameters of the Gait profile score (GPS) [29].

### Statistical analysis

Descriptive statistics are presented as medians and interquartile ranges (IQR) (Table 1). Nonparametric analyses were applied due to the small sample sizes and variability observed within each cohort. Longitudinal changes in spatio-temporal, postural control, and anthropometric parameters were analyzed using the R package nparLD for nonparametric analysis of longitudinal data, employing the F1-LD-F1 model and ANOVA-type statistics (ATS) [30]. Kinematic parameters were quantified using the GPS and Gait Variable Score (GVS), with longitudinal changes analyzed using the F1-LD model and ATS within nparLD. This rank-based approach provides a robust method suitable for small sample sizes [30]. Statistical significance was set at *p* = 0.05.

Effect sizes for the main effects in nparLD were estimated using the relative treatment effect (RTE), where RTE > 0.5 indicates a difference favoring the ACH group, RTE < 0.5 favors the CAH group, and RTE = 0.5 indicates no difference. Post hoc effect sizes were calculated using the r statistic, with thresholds of 0.1, 0.3, and 0.5 representing small, medium, and large effects, respectively [31]. All statistical analyses were performed in R [32].

To evaluate whether observed changes between measurement time points reflected systematic rather than random changes, the smallest real difference (SRD) was calculated following Lexell and Downham [33] using the equation: -$\mathrm{SRD}\:=1.96\:x\:SEM\:x\:\sqrt{2}$. The Standard Error of Measurement (SEM) was derived from a robust standard deviation (SD_r_), calculated as $\:SDr=Median\:Absolute\:Error\:x\:1.4826\:$, with $\:SEM=SDr\:x\:\sqrt{(1}-Rho)$. Given the nonparametric nature of the data, Spearman’s Rho was used for correlations.

### Posturographic parameters

CoP raw data in all three axes from the force plate was exported as txt.files through Vicon Nexus. With a customized MATLAB Code (Version 25.2.0.3042426 (R2025b), Natick, MA, USA, TheMathWorks Inc.), data was processed using a low pass 5th order Butterworth filter with a cut-off frequency of 10 Hz. Following parameters were calculated: length of CoP (mm) (anterior - posterior (AP) and medio – lateral (ML)), sway area (mm^2^), and the frequency in three defined ranges (low (LF) 0.02 Hz up to 0.1 Hz, medium (MF) 0.1 Hz up to 1 Hz and high (HF)1 Hz up to 10 Hz). Those ranges were defined after the protocol of Fujimoto et al. [26], given that above 10 Hz the motor units in the soleus muscle can synchronize at an interval of at least 100 ms during quiet standing [26]. Furthermore, Power Spectral Density (PSD) was calculated to define how long each participant stayed in total in each frequency range, and the log of this PSD was used to analyze the data. The decision to use the log was based on Watanabe et al. [27] because in the low-frequency area, the curves are often steep and not appropriate to visualize in a good manner without using a log. Using PSD, the area under the curve (AUC) for each frequency range was calculated and used for further statistical analysis.

## Results

**Fig. 1:**
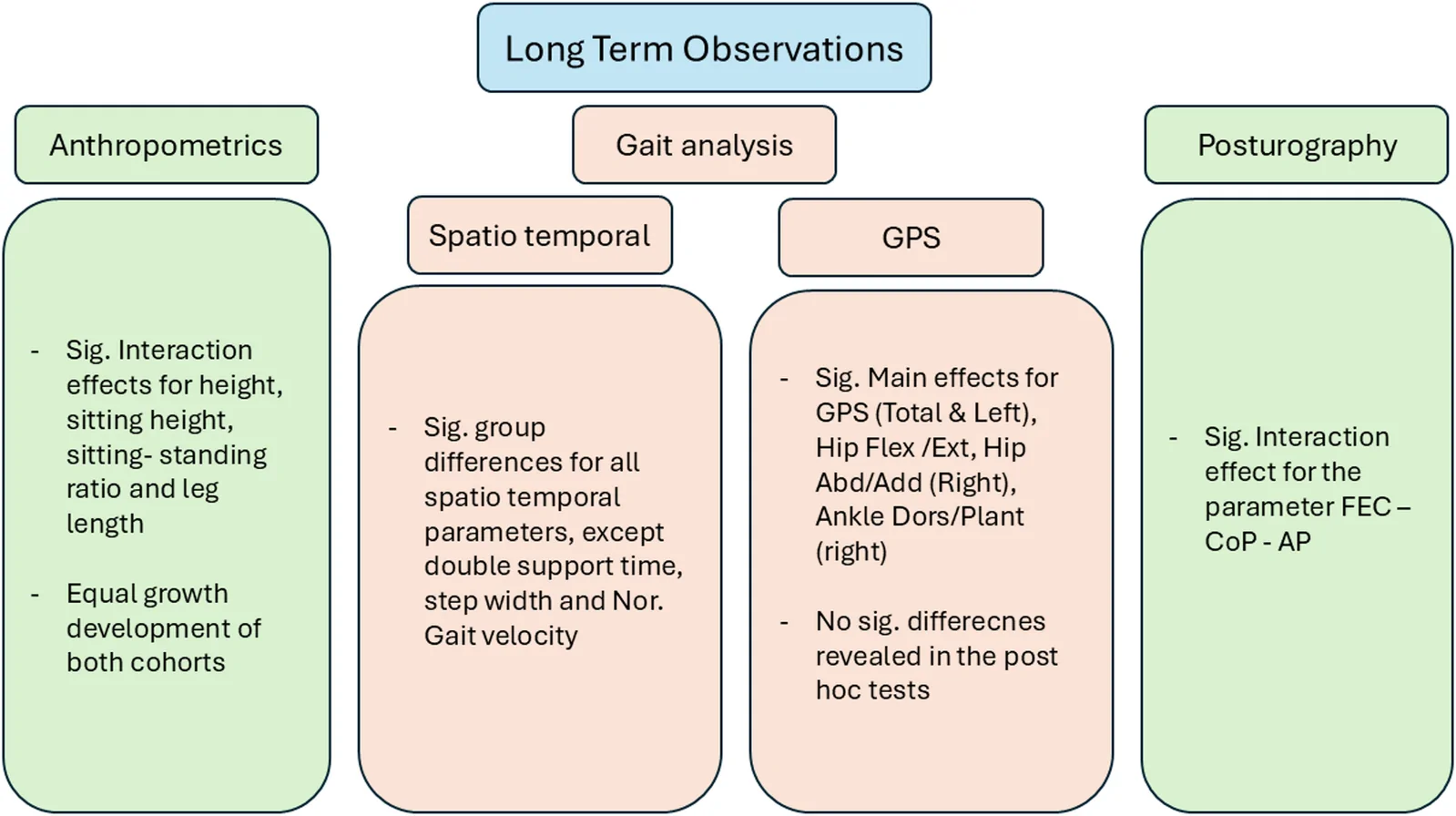
Overview of the results of the current study. ACH = Achondroplasia. SRD = Small real difference. EC - Eyes closed, CoP = Center of pressure, AP = Anterior posterior, LF = Low frequency, FEO = Foam pad eyes open. GPS = Gait profile score

Results are briefly presented as an overview in Fig. 1 and described in detail in the following section.

### Anthropometrics

Descriptive statistics of the anthropometric parameters are presented in Table 1 as median and IQR and changes over time are visualized in Supplementary File (SF) SF Fig. 1.

#### F1 – LD – F1 Model

Significant interaction effects were observed for the parameters height (*p* = 0.01), sitting height (*p* = 0.01), sitting-standing ratio (*p* < 0.001) and leg length (*p* < 0.001).

Besides significant group differences were found for the parameters FLR (all four timepoints *p* < 0.001, *r* = 0.68) and pelvic tilt (all four timepoints *p* < 0.001, *r* = 0.39–0.61). Significant time differences for CAH were additionally found for weight (all three timepoints *p* < 0.001, *r* = 0.76–0.87) and age (all three timepoints *p* < 0.001, *r* = 0.87).

### Spatio temporal parameters

Changes over time for all children with ACH and are presented in SF Figs. 2 and 3. No significant interaction effects were observed.

#### F1 – LD – F1 Model

Significant main effects for the factor group were shown for all parameters (*p* = < 0.001–0.04) except double support time and normalized gait velocity Post hoc tests shown significant differences at all four time points for the parameters single support time (p = < 0.001–0.01, *r* = 0.49–0.65), stride (*p* < 0.001, *r* = 0.66–0.68) & step length (*p* < 0.001, *r* = 0.66–0.68) and normalized cadence (*p* < 0.001, *r* = 0.54–0.63). Cadence revealed significant differnces at T1 (*p* = 0.02, *r* = 0.51). Stride time is significantly different at T1 (*p* = 0.01, *r* = 0.50) as well as step time at T1 (*p* = 0.02, *r* = 0.51). Stance and swing phase have significant differences at T1 (*p* = 0.02, *r* = 0.51) and T4 (*p* = 0.01, *r* = 0.56). Furthermore, significant main effects for the factor time (Table SF 2) were revealed for the parameters cadence (*p* = 0.02), stride (*p* = 0.01), single support time (*p* < 0.001), stride (*p* < 0.001) & step length (*p* < 0.001). Post hoc tests revealed for CAH significant differences for the parameter single support time at time point T2 – T3 (*p* = 0.03, *r* = 0.54) and time point T3 – T4 (*p* = 0.03, *r* = 0.51) and the parameter stride length at T2 – T3 (*p* = 0.02, *r* = 0.61).

#### SRD

ACH presents changes for the SRD for different parameters and different points of time (see Table SF 10) for the spatio temporal parameters. Table 2  provides an overview of all crossings of SRD for each participant at each time point.

**Table 2:** Systematic changes for all spatio-temporal parameters based on the SRD between the different time points presented for every child with ACH. T1 = Time point 1, T2 = Time point 2, T3 = Time point 3, T4 = Time point 4, ACH = Achondroplasia

Parameter	Measurement Point		
	T1 - T2	T2 - T3	T3 - T4
Gait velocity	P1,P4		
Cadence	P1		
Step time	P1	P4,P6	P1
Stride time	P1	P4,P6	
Single support time	P1	P1,P6	
Double support time		P4	
Stance phase	P4		
Swing phase	P4		
Stride length		P3	
Step length	P5	P3	
Step width		P1,P2	P6
Normalized cadence	P1		
Normalized gait velocity			
Normalized step length			
Normalized stride length			

#### GPS

The GPS were calculated only for ACH based on the reference cohort as part of the calculation of the GPS. Therefore, only the factor time in the model F1 – LD can be considered. All overtime changes are presented in SF Figs. 4 and 5.

#### F1 – LD - Model

Significant main effects for the factor time (Table SF 5) were revealed for the parameters GPS (*p* < 0.001), GPS Left (*p* = 0.04), Hip Flex/Ext Left (*p* < 0.001), Hip Flex/Ext Right (*p* < 0.001), Hip Abd/Add Right (*p* < 0.001) and Ankle Dors/Plantarflexion Right (*p* = 0.02). No significant differences were revealed through the post hoc tests.

#### SRD

Table SF 11 presents an overview of all the GPS related parameters and their SRD values and Table 3  reveals the surpassing of the SRD at each time point for each participant.

**Table 3:** Systematic changes for all GPS related parameters based on the SRD between the different time points presented for every child with ACH. T1 = Time point 1, T2 = Time point 2, T3 = Time point 3, T4 = Time point 4, ACH = Achondroplasia

Parameter	Measurement Point		
	T1 - T2	T2 - T3	T3 - T4
GPS	P1 – P6	P5	
GPS (L)	P4-P6		
Hip Flex/Ext (L)	P4	P1,P2	P1
Hip Abd/Add (L)	P4	P5	
Hip Rotation (L)	P4	P4	
Knee Flex/Ext (L)	P2,P3		
Ankle Dors/Plantarflex (L)	P2	P1,P3	
Foot Progression (L)			
GPS (R)	P1,P3	P5	
Hip Flex/Ext (R)	P3 – P5	P1,P2,P4,P5	
Hip Abd/Add (R)	P5	P1,P3,P5	
Hip Rotation (R)	P2		P2
Knee Flex/Ext (R)			P3
Ankle Dors/Plantarflex (R)		P2,P4,P5	P6
Foot Progression (R)	P1 – P6		P1 – P6
Pelvic Tilt			P1
Pelvic Obliqutiy		P1,P6	P1,P3,P4,P5
Pelvic Rotation	P1 – P6	P1	P5

### Posturographic parameters

All posturographic parameters are presented as well in SF Figs. 6, 7, 8, 9 and 10 and the SRD values are listed in Table SF 12 and SF 13. For the comparison between cohorts and the observation over time the F1 – LD – F1 Model was used again. It must be noted that for those parameters only five children could be evaluated due to missing data for one child (P3). However, the order of numbers stays persistent to previous descriptions.

### CoP AP, CoP ML & Sway

#### F1 – LD -F1 Model

Significant interaction effect was observed for the parameter FEC – CoP- AP (*p* = 0.04). However, despite a significant main effect for the factor group, parameter EO – CoP – AP (*p* = 0.03, RTE ACH = 0.70, RTE CAH = 0.45), no significant post hoc differences were revealed.

#### SRD

All results of the SRD regarding the parameters CoP AP, CoP ML and Sway can be found in Table SF 12. Also here, Table 4  reveals the surpassing of the SRD at each time point for each participant.

**Table 4:** Systematic changes for all CoP parameters based on the SRD between the different time points presented for every child with ACH. T1 = Time point 1, T2 = Time point 2, T3 = Time point 3, T4 = Time point 4, ACH = Achondroplasia

Parameter	Measurement Point		
	T1 - T2	T2 - T3	T3 - T4
EO			
CoP AP	P1 – P6	P1,P2	
CoP ML			
CoP Sway	P2	P1,P2	
EC			
CoP AP	P2	P2	P1 – P6
CoP ML	P2	P2	P2
CoP Sway	P2 – P6	P1,P2	P2,P4
FEO			
CoP AP	P6		P1 – P6
CoP ML	P1,P4,P5,P6	P1 – P6	P5, P6
CoP Sway		P1 – P6	P1 – P6
FEC			
CoP AP	P1	P1 – P6	P2,P4,P6
CoP ML	P2	P1 – P6	P1 – P6
CoP Sway	P2,P5	P2,P4	P2,P4

### AUC Parameters

#### F1 – LD -F1 Model

Significant main effects for the factor Group (SF Table 8) were revealed for the condition EC and FEO. For the condition EC with the parameter LF – AP (*p* = 0.01, RTE ACH = 0.25, RTE CAH = 0.56) and for the condition FEO – with the parameter LF - AP (*p* = 0.01, RTE ACH = 0.12, RTE CAH = 0.59) and LF – ML (*p* = 0.03, RTE ACH = 0.36, RTE CAH = 0.53). Post hoc tests revealed for EC – LF – AP significant differences (*p* = 0.01, *r* = 0.65) at T1 and for FEO – LF – AP for all four time points (*p* < 0.001–0.01, *r* = 0.51–0.65).

#### SRD

All results of the SRD regarding the AUC parameters can be found as well in Table SF 13 and Table 5  presents the crossinf of the SRD for each participant and each time point.

**Table 5:** Systematic changes for all AUC parameters based on the SRD between the different time points presented for every child with ACH. T1 = Time point 1, T2 = Time point 2, T3 = Time point 3, T4 = Time point 4, ACH = Achondroplasia

Parameter	Measurement Point		
	T1 - T2	T2 - T3	T3 - T4
EO			
LF - AP		P4	P1 – P6
MF - AP			P4
HF - AP	P1 – P6	P1,P2	
LF - ML	P2,P4,P6	P1	P1
MF - ML	P4,P6	P1	
HF - ML			
EC			
LF - AP	P1 – P6		P4
MF - AP	P2 – P6	P2,P4	P4
HF - AP	P2,P6		P2
LF - ML	P2,P6		
MF - ML	P2,P6	P1,P2	P2
HF - ML	P2,P6	P2	P2
FEO			
LF - AP		P2	P2,P4,P6
MF - AP		P4	P1 – P6
HF - AP	P5,P6	P1 – P6	P2
LF - ML		P2	P1,P2,P4,P6
MF - ML	P6		P1
HF - ML	P4 – P6	P6	P1 – P6
FEC			
LF - AP	P2,P5	P2,P4	P2,P4
MF - AP	P2,P4,P5	P4	P4
HF - AP	P1,P2	P2,P4	P1 – P6
LF - ML	P2,P5	P6	P2
MF - ML	P2,P5	P1,P2,P4	P2,P4
HF - ML	P2	P2	P2

## Discussion

This study aimed to investigate changes in gait patterns and static posturographic abilities in children with ACH over a two-year period. Non-parametric statistical methods, including nparLD and the SRD, were applied to evaluate these changes. Due to the final small sample size and heterogeneity of the participants, the focus shifted from statistics to primarily on effect sizes in order to highlight tendencies within the observed parameters. The authors are aware of the limitations associated with the small sample and the resulting statistical constraints.NpardLD was selected to diminish the contraints as well as possible. However, as this study is lastly intended as a pilot investigation, statistical methods were selected that are theoretically appropriate and suitable for analyses in future studies with larger sample sizes. The anthropometric parameters confirmed the differences between both cohorts knowing already from literature [7, 8]. Knowing the shorter stature, increased pelvic tilt and shorter leg length as well as the differences for the ratios of standing to sitting and foot to leg, those results were as expected. However, looking at the factor time of the F1 – LD – F1 Model, the post hoc test revealed no significant differences, indicating an identical growth pattern for both cohorts. Still, most of the parameters for both cohorts revealed strong effect sizes, underlining again the identical growth pattern. This might be affected by the fact that all children with ACH, except P6, are treated with a Vosoritide. Vosoritide has a positive effect on growth, especially the long bones of the arms and legs. Unfortunately, we have no cohort without the treatment to investigate this effect more. Still, we can link our results to the literature. In a recent study we could confirm an increased growth velocity in real world study [21], in line with the observations of 5.75 cm after 3 years in the approval study for Vosoritide [34].This translates to a similar growth rate pattern as CAH [21]. SF Fig. 1 shows the changes over time for all seven children with ACH, all parameters show a linear growth pattern despite the two ratio parameters. Those display quite some variability, which might be due to different growth of body parts. For the pelvic tilt as well, an increase is appearing over time, which is for children with ACH a risk for spinal stenosis and back pain. This is contradictonary to the results of vosoritide treatment of Sawamura et al. [22], who stated a slightly positive change of the spine, yet it has to be considered that we cannot compare to a natural development of the pelvic for children with ACH. It might be that the increase of the pelvic tilt is decelerated.

Moving to the spatio-temporal parameters, linked to the knowledge from previous studies, the differences between the cohorts were not surprising [6, 12–15]. The decreased gait velocity and increased cadence, whereby the latter is still with significant changes after normalization, as well as the decreased step and stride length are known. Besides significant differences, all of those parameters are revealing great effect sizes, emphasizing those differences again. Through normalization, those effects are diminishing for all parameters except cadence, underlining the influence of the leg length. Furthermore, a slightly delayed foot off at the end of the stance phase was present, resulting in a significantly different swing phase. This longer duration of the stance phase, leading to a shorter swing phase, might be an explanation for the significant changes of the single support time. However, greater effects were occuring for CAH at T2-T3 and T3-T4. Underlining greater variability for this group during single support time. Still it is theorized that challenges to maintain unipedal balance might be a reason for the shorter single support time for some children with ACH This theory is based on observation during the measurements. However, no research has been done on this aspect until now. Regarding the changes over time, no significant differences occurred for ACH, indicating for a normal growth-related developmental pattern of those parameters. Investigating the SRD for ACH, for some participants a variability in the gait pattern is present, whereas others seem to present a quite stable gait pattern. E.g. P1 presents multiple changes from T1 to T2, but for the other two comparisons of time points seem to stabilize and barely present any systematic changes anymore. Additionally, P4 shows at T1 - T2 and T2 – T3 great variability. Most systematic changes are occurring for the temporal parameters. Schwartz et al. [35] underline the importance of the speed during walking and its effects on the kinematics and kinetics. Still as well we need to consider that the changes occurring between the measurements might be related to the ongoing development of the gait pattern. Known from literature up to going into adolescents children still are developing their gait pattern [1]. Additionally, it is also known that for ACH slight delays in motor development can occur [7], which might further explain those variabilities. Lastly, we need to consider that the children walked with a self-selected walking speed, that can change due to many different factors. For future studies with greater sample sizes both aspects need to be considered, while evaluating the data.

Regarding the kinematic parameters, we decided to investigate the changes over time using the GPS [29]. The GPS is a score that evaluates 18 selected kinematic parameters, for which every parameter’s GVS is calculated and lastly calculates the GPS out of it [29]. Here, only changes over time were considered, due to the calculation process of the GPS and the fact that CAH served as reference score for ACH. Unfortunalety, the GPS only includes kinematic parameters. No valid kinetic equivalent is accesible for the authors. Due to that and the great number of results presented here, it was decided to exclude kinetics.Still, future research should target that gap. The F1-D-Model could not reveal any significant differences over time after computing the post hoc tests. However, great effect sizes were visible for some parameters, strongly seen for the parameters of the hip and the GPS, general and both sides individually. Linking the results of the F1 – LD Model to the SRD, the variability in the temporal parameters and the age of the children, those great effects might be easier to understand.

P1 and P4 revealed systematic changes for most of the temporal parameters. Both children show changes as well for the kinematic parameters, which might be a result from that. P1 additionally is a younger child. P4 a more inactive and obese child. Those are additonally factors that might explain the variability.Generally, more systematic changes are seen for the kinematic parameters. Strongly seen in the hip parameters, followed by the pelvic parameters. It is known that Vicon struggles to calculate the hip joint center adequate for people with ACH [36]. This might explain this variability. Currently, the work group around Hösl [37] aims to develop a robust algorithm to calculate the center in respect to the malalignments.

Lastly, relating to the posturographic parameters and the AUC parameters, barely significant differences were revealed in the post hoc tests. A significant interaction effect was observed for the paramer FEC- CoP – AP.However, looking at the group and time factor, no significant differences occurred. Still, the RTE indicates for the comparison between the groups, that children with ACH show a tendency to greater values for almost all parameters. Interestingly for the post hoc tests, barely medium effect sizes occur. Additionally, only EO – CoP – AP (*r* = 0.67) and FEC – CoP- AP (*r* = 0.53) for CAH reveal great effect sizes. This paradox occurrence might be a result of the small sample size of children with ACH compared to CAH, affecting the statistics. The absence of significant differences is in agreement with a previous pilot study of this research group [5]. Likewise, only significant differences occurred for the parameter FEC - CoP – AP.

Regarding the AUC parameters, for the factor group only two AUC parameter show significant differences. EC - LF – AP differs significantly T1 & T2. Equally FEO – LF – AP at all four time points reveals significant differences. Still, great effect sizes are occuring for multiple paramters in the LF range for the AP direction (see SF Tables 8 and 9) This appearance is present for both factors, with a greater occurrence for children with ACH for the factor time. However, it must be mentioned that while there are barely significant differences for either the factor time or group, the variability inside the cohort was tremendous. A seen in Table SF 12 and SF 13 and SF Figs. 6, 7, 8, 9 and 10e.g. P2 presents greater values for all parameters. It can be here discussed to label a child such as this as an outlier and exclude it from the study. The authors decided against it after confirming that there are no significant changes in the results with that child. This decision was made due to the small sample size and furthermore to display the variety of the disability. Additionally, most of the systematic changes occurred during eyes closed conditions. Knowing that balance is based on a multi sensory feedback system [2], it seems that the vision is strongly affecting the postural control of children with ACH.

In conclusion, although previous studies have not consistently demonstrated general static balance deficits or an increased risk of falls in individuals with ACH, features such as anterior pelvic tilt, hypotonia, and joint hyperlaxity may influence postural control in children with ACH, potentially contributing to the manifestation of functional limitations. Overall, the present findings are largely consistent with prior work from this research group [5]. Frequency analyses did not reveal a clear pattern favoring either group. Considering the RTE, children with ACH exhibited greater effects under EO and EC conditions, whereas under FEO and FEC, effects shifted in favor of CAH. With respect to anthropometric parameters and the observed increase in pelvic tilt over time, posturographic measures appeared to stabilise in some children, whereas in others, a slight progression persisted. The factors underlying these individual differences remain unclear, though activity levels and participation in specific sports may positively influence postural control outcomes. Table 6 displays the physical activity of all ACH during a week. The differences between them are quite tremendous, going from four different sports a week down to only home-based strength training without active participation in schools’ gym class. P1 – P4 participating in less physical activity, which could be a reason for the greater variability and greater results compared to the other ACH. Furthermore, the different sports and their demands on the postural control might affect it. Either way research needs to be done on that topic to confirm our assumptions.

### Clinical implications of the study

Overall, the results of this study state clearly that there are no general differences in the growth development between treated children with ACH and CAH. However, the baseline of both cohorts has to be considered as well as the fact of the treatment of close to all children with ACH (see Table 7). Children with ACH have due to their characteristics of the disability an already changed gait pattern compared to CAH. During the growth this pattern will develop in respect to those characteristics. Yet, Sawamura et al. [23] stated after a year of treatment positive effects on the spine and knee malalignments. Therefore, the pelvic and knee parameters should be considered as important parameters to be included in the evaluation. Especially to see the differences between treated and untreated children.

The use of dynamic long-term observations such as with 3DGA should be mandatory to observe and evaluate patients. However, due to the heterogeneity of this cohort, the use of single case studies would be more benefical for medical staff to observe the individual developtment of a child.

Furthermore, the pronouncement of ACH, physical activity and cormorbidities need to be considered and investigated. Until now, no studies about the positive effect of physical activity on the gait pattern exist. Alves et al. [19] found moderate to strong positive correlation between sports and postural control for adults with ACH, providing a basline for future research.

### Limitations of the study and future research directions

This study has several limitations that should be considered. First, the study population consisted of a small and unbalanced sample, with only six children ACH and 24 CAH completing the study. Initially, 16 children with ACH were enrolled, but 10 dropped out, primarily due to the long travel distance to the gait laboratory (approximately 5 km) and low motivation to participate. Despite these dropouts, the larger control group provided a robust reference for GPS calculations. For posturographic analyses, only five children with ACH were included due to technical errors with the force plate for one participant. The small sample size reflects both the rarity of ACH and the challenges associated with recruiting participants externally through medical appointments, which often require families to travel long distances and take time off work. Participation in long-term studies is challenging, and no financial compensation was available, despite efforts to retain participants throughout the study.

Second, results based on the SRD should be interpreted with caution due to the small sample size. Lexell and Downham [33] recommend a minimum of 30–50 participants for meaningful SRD estimates; however, most comparable studies report 15–20 participants. Consequently, statistical interpretations in this study should be viewed as preliminary and primarily illustrative of potential approaches for future investigations with larger cohorts.

Third, kinetic parameters could not be analyzed for children with ACH due to limitations of the GPS, which restricts calculations to specific kinematic measures. Future studies investigating kinetics would be valuable, particularly given that lower-limb malalignments may increase the risk of joint degeneration and early-onset arthrosis.

Thirdly, we could not investigate kinetic parameters for ACH, due to limitations of the GPS, where only specific parameters are usable as a result of the foundation of the calculations. This would be for further research an interesting aspect, given that the malalignments create higher risks for joint erosion, resulting in earlier arthrosis risks.

## Conclusion

This study provides a methodological framework for long-term evaluation of gait and postural control in children with ACH, which may also be applicable to other rare conditions. Despite the extensive data collected, several questions remain, including the use of unipedal balance tasks and long-term kinetic assessments. Future research should explore broader age ranges, potential gender differences, untreated populations, and larger sample sizes, potentially through multicenter collaborations. These findings highlight the value of single-case analyses in rare diseases, as individual variability in ACH is substantial and may be obscured by group-level comparisons.

**Table 6:** Physical activity of all children with ACH. ACH = Achondroplasia. * = Badminton played on elite athlete level. ** = elite level athlete camp

Patient		Activity	Duration	Activity	Duration	Activity	Duration	Activity	Duration
	Sex		(h/week)		(h/week)		(h/week)		(h/week)
P1	w	Horse Riding	3 × 1/2	Gym class	2				
P2	m	Swimming	1	Gym class	1 1/2				
P3	w	Horse Riding	3 × 1/2	Gym class	1 1/2				
P4	m	Body Weight Strength Training	7 × 0.25	Strength Training	0.75 *				
P5	w	Horse Riding	1 1/2	Dancing	1 × 2	Popular sports	1 1/2		
P6	w	Badminton*	3 × 1–1 1/2	Gym class	1 1/2	Performance course**	whole week	Physiotherapy	0.3

**Table 7:** Treatment duration of Vosoritide for P1 - P5. P6 did not take the drug

Patient	Sex	Treatment
P1	w	3.38 years
P2	m	3.76 years
P3	w	3.88 years
P4	m	3.58 years
P5	w	2.02 years

## Supplementary Information

Supplementary Material 1.

## Data Availability

The dataset supporting the conclusions of this article is included within the article (and its additional file(s)).

## Abbreviations

ACH: Achondroplasia
CAH: Children of average height
CoP: Center of pressure
3DGA: Three-dimensional gait analysis
EO: Eyes open
EC: Eyes closed
FEO: Foampad eyes open
FEC: Foampad eyes closed
AP: Anterior – posterior
ML: Medio – lateral
LF: Low frequency
MF: Medium frequency
HF: High frequency
PSD: Power Spectral Density
AUC: Area under the curve
GPS: Gait profile score
IQR: Interquartile range
ATS: ANOVA type statistics
GVS: Gait variable score
RTE: Relative treatment effect
SEM: Standard error of measurement
SRD: Smallest real differences
SDr: Robust Standard Deviation

## References

[1] Lythgo N, Wilson C, Galea M. Basic gait and symmetry measures for primary school-aged children and young adults. II: walking at slow, free and fast speed. Gait Posture. 2011;33:29–35. 10.1016/j.gaitpost.2010.09.017. 20971013 10.1016/j.gaitpost.2010.09.017

[2] Gouleme N, Ezane MD, Wiener-Vacher S, Bucci MP. Spatial and temporal postural analysis: a developmental study in healthy children. Int J Dev Neurosci. 2014;38:169–77. 10.1016/j.ijdevneu.2014.08.011. 25196999 10.1016/j.ijdevneu.2014.08.011

[3] Armand S, Moissenet F, de Coulon G, Bonnefoy-Mazure A. Identifying and understanding gait deviations: critical review and perspectives. Mov Sport Sci/Sci Mot. 2017;77–88. 10.1051/sm/2017016.

[4] García-Soidán JL, Leirós-Rodríguez R, Romo-Pérez V, García-Liñeira J. Accelerometric Assessment of Postural Balance in Children: A Systematic Review. Diagnostics (Basel). 2020. 10.3390/diagnostics11010008. 33375206 10.3390/diagnostics11010008 PMC7822105

[5] Hergenröther M, Palm K, Mohnike K, Witte K. Bipedal static postural stability in children with achondroplasia compared to typically developed children in the age range of 9–12 years - A pilot study. Gait Posture. 2025;117:337–41. 10.1016/j.gaitpost.2025.01.005. 39842157 10.1016/j.gaitpost.2025.01.005

[6] Broström EW, Antonissen L, von Heideken J, Esbjörnsson A-C, Hagenäs L, Naili JE. Gait in children with achondroplasia - a cross-sectional study on joint kinematics and kinetics. BMC Musculoskelet Disord. 2022;23:397. 10.1186/s12891-022-05343-4. 35484571 10.1186/s12891-022-05343-4 PMC9047292

[7] Pauli RM. Achondroplasia: a comprehensive clinical review. Orphanet J Rare Dis. 2019;14:1. 10.1186/s13023-018-0972-6. 30606190 10.1186/s13023-018-0972-6 PMC6318916

[8] Merker A, Neumeyer L, Hertel NT, Grigelioniene G, Mohnike K, Hagenäs L. Development of body proportions in achondroplasia: Sitting height, leg length, arm span, and foot length. Am J Med Genet A. 2018;176:1819–29. 10.1002/ajmg.a.40356. 30152086 10.1002/ajmg.a.40356

[9] Ain MC, Shirley ED, Pirouzmanesh A, Skolasky RL, Leet AI. Genu varum in achondroplasia. J Pediatr Orthop. 2006;26:375–9. 10.1097/01.bpo.0000203013.04272.b6. 16670552 10.1097/01.bpo.0000203013.04272.b6

[10] Egginton R, Newman C, Walsh M, Jenkinson A, Bennett D, O’Brien T. Kinematic characteristics of Achondroplasia. Gait Posture. 2006;24:S249–50. 10.1016/j.gaitpost.2006.11.170.

[11] Hösl M, Thamm A, Afifi FK, Nader S. Frontal Plane Knee Kinematics and Kinetics During Gait in Children and Youth with Achondroplasia-Correspondence with Static X-Ray Images and Relevance to Symptoms. Child (Basel). 2025. 10.3390/children12010078. 10.3390/children12010078 PMC11763588 39857909

[12] Kiernan D. Lower limb biomechanics during gait in children with Achondroplasia. J Biomech. 2021;119:110313. 10.1016/j.jbiomech.2021.110313. 33631664 10.1016/j.jbiomech.2021.110313

[13] Sims DT, Burden A, Payton C, Onambélé-Pearson GL, Morse CI. A spatio-temporal and kinematic description of self-selected walking in adults with Achondroplasia. Gait Posture. 2020;80:391–6. 10.1016/j.gaitpost.2020.06.030. 32650227 10.1016/j.gaitpost.2020.06.030

[14] Sims DT, Burden A, Payton C, Onambélé-Pearson GL, Morse CI. A quantitative description of self-selected walking in adults with Achondroplasia using the gait profile score. Gait Posture. 2019;68:150–4. 10.1016/j.gaitpost.2018.11.019. 30476692 10.1016/j.gaitpost.2018.11.019

[15] Inan M, Thacker M, Church C, Miller F, Mackenzie WG, Conklin D. Dynamic lower extremity alignment in children with achondroplasia. J Pediatr Orthop. 2006;26:526–9. 10.1097/01.bpo.0000217712.42115.e6. 16791073 10.1097/01.bpo.0000217712.42115.e6

[16] Karikari IO, Mehta AI, Solakoglu C, Bagley CA, Ain MC, Gottfried ON. Sagittal spinopelvic parameters in children with achondroplasia: identification of 2 distinct groups. J Neurosurg Spine. 2012;17:57–60. 10.3171/2012.3.SPINE11735. 22540171 10.3171/2012.3.SPINE11735

[17] Cai H, Omara C, Castelein R, Vleggeert-Lankamp CL. Sagittal balance parameters in achondroplasia. Brain Spine. 2023;3:102670. 10.1016/j.bas.2023.102670. 38021024 10.1016/j.bas.2023.102670 PMC10668104

[18] Hong J-Y, Suh S-W, Modi HN, Park J-W, Park J-H. Analysis of sagittal spinopelvic parameters in achondroplasia. Spine (Phila Pa 1976). 2011;36:E1233–9. 10.1097/BRS.0b013e3182063e89. 21358483 10.1097/BRS.0b013e3182063e89

[19] Alves I, Castro MA, Tavares S, Fernandes O. Unveiling the Chaos in Postural Control in Adults with Achondroplasia. J Funct Morphol Kinesiol. 2024. 10.3390/jfmk9010039. 38535419 10.3390/jfmk9010039 PMC10970844

[20] Yin J, Cong W, Wang Y, Zhou C. Three-dimensional gait analysis in spinal disorders: biomechanical insights and clinical applications for diagnosis, surgical planning, and rehabilitation. Front Neurol. 2025;16:1666267. 10.3389/fneur.2025.1666267. 41127291 10.3389/fneur.2025.1666267 PMC12537708

[21] Mohnike K, Beger C, Gausche R, Hoyer-Kuhn H, Muschol N, Palm K, et al. The CrescNet Registry Achondroplasia Module: Real-World Demographic Data and Clinical Outcomes in Untreated and Vosoritide-Treated Individuals. Horm Res Paediatr. 2026;1–24. 10.1159/000550795. 10.1159/000550795 PMC13046303 41615893

[22] Albuquerque ALB, Dacoregio MI, Rodrigues CG, Bertola DR, Zattar Ribeiro PV. Real-world outcomes of vosoritide in achondroplasia: A systematic review and meta-analysis of multinational clinical evidence. Genet Med. 2026;28:101670. 10.1016/j.gim.2025.101670. 41424367 10.1016/j.gim.2025.101670

[23] Sawamura K, Kitoh H, Kamiya Y, Mishima K, Matsushita M, Imagama S. Changes in the Alignment of the Spine and Lower Limb in Children With Achondroplasia Treated With Vosoritide: A Single-center, 1-year Follow-up Prospective Study. J Pediatr Orthop. 2025;45:519–24. 10.1097/BPO.0000000000002980. 40231376 10.1097/BPO.0000000000002980

[24] Stief F, Böhm H, Michel K, Schwirtz A, Döderlein L. Reliability and accuracy in three-dimensional gait analysis: a comparison of two lower body protocols. J Appl Biomech. 2013;29:105–11. 10.1123/jab.29.1.105. 22813723 10.1123/jab.29.1.105

[25] Cha E, Park JH. Spinopelvic Alignment as a Risk Factor for Poor Balance Function in Low Back Pain Patients. Global Spine J. 2023;13:2193–200. 10.1177/21925682221076417. 35220778 10.1177/21925682221076417 PMC10538310

[26] Fujimoto C, Kamogashira T, Kinoshita M, Egami N, Sugasawa K, Demura S, et al. Power spectral analysis of postural sway during foam posturography in patients with peripheral vestibular dysfunction. Otol Neurotol. 2014;35:e317–23. 10.1097/MAO.0000000000000554. 25111526 10.1097/MAO.0000000000000554

[27] Watanabe H, Makabe A, Hirai C, Takeda T, Honda K, Demura S, Tsutsumi T. Frequency analyses of posturography using logarithmic translation. Acta Otolaryngol. 2020;140:1–7. 10.1080/00016489.2019.1682660. 31709868 10.1080/00016489.2019.1682660

[28] Hof AL. Scaling gait data to body size. Gait Posture. 1996;4:222–3. 10.1016/0966-6362(95)01057-2.

[29] Baker R, McGinley JL, Schwartz MH, Beynon S, Rozumalski A, Graham HK, Tirosh O. The gait profile score and movement analysis profile. Gait Posture. 2009;30:265–9. 10.1016/j.gaitpost.2009.05.020. 19632117 10.1016/j.gaitpost.2009.05.020

[30] Noguchi K, Gel YR, Brunner E, Konietschke F. nparLD: An R Software Package for the Nonparametric Analysis of Longitudinal Data in Factorial Experiments. J Stat Soft. 2012. 10.18637/jss.v050.i12.

[31] Cohen J. Statistical Power Analysis for the Behavioral Sciences. Routledge; 2013.

[32] R Core Team. R: A language and environment for statistical. Vienna, Austria: R Foundation for Statistical Computing; 2025.

[33] Lexell JE, Downham DY. How to assess the reliability of measurements in rehabilitation. Am J Phys Med Rehabil. 2005;84:719–23. 10.1097/01.phm.0000176452.17771.20. 16141752 10.1097/01.phm.0000176452.17771.20

[34] Savarirayan R, Irving M, Wilcox WR, Bacino CA, Hoover-Fong JE, Harmatz P, et al. Sustained growth-promoting effects of vosoritide in children with achondroplasia from an ongoing phase 3 extension study. Med. 2025;6:100566. 10.1016/j.medj.2024.11.019. 39740666 10.1016/j.medj.2024.11.019

[35] Schwartz MH, Rozumalski A, Trost JP. The effect of walking speed on the gait of typically developing children. J Biomech. 2008;41:1639–50. 10.1016/j.jbiomech.2008.03.015. 18466909 10.1016/j.jbiomech.2008.03.015

[36] Broström E, Gutierrez-Farewik EM, Örtqvist M, Hagenäs L, Neumeyer L, Rozumalski A, Schwartz MH. A comparison of functional and regression-based hip joint centers in persons with achondroplasia. Gait Posture. 2009;30:S81–2. 10.1016/j.gaitpost.2009.08.121.

[37] Hösl M, Thamm A, Nader S. Frontal plane X-ray-based regression equation for more accurate hip joint center localization in gait analysis of children with Achondroplasia. Gait Posture. 2025;121:92–3. 10.1016/j.gaitpost.2025.07.103.

