# Supplementary material for: Gait and posturographic parameters of children with achondroplasia – a long-term pilot study
Source: BMC Musculoskelet Disord. 2026 Apr 2;27:304. doi: 10.1186/s12891-026-09780-3 (PMC13069793; doi:10.1186/s12891-026-09780-3)
Supplement: Supplementary file 1 — Supplementary Material 1. [file 12891_2026_9780_MOESM1_ESM.docx]

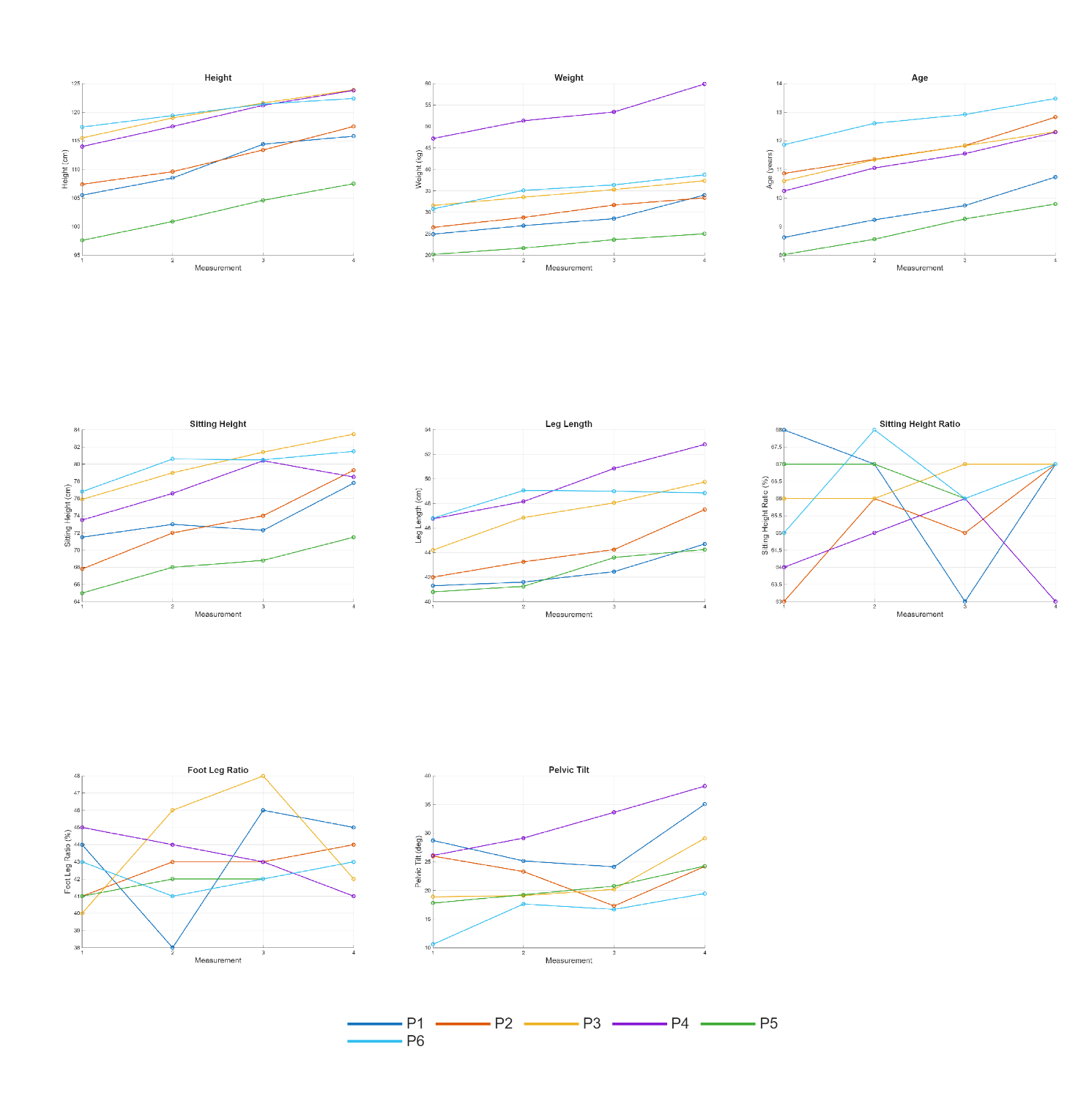
Supplementary Files

Figure SF 1 Anthropometric parameters for all children with ACH for all four time points to display the individual development of each child. ACH = Achondroplasia.


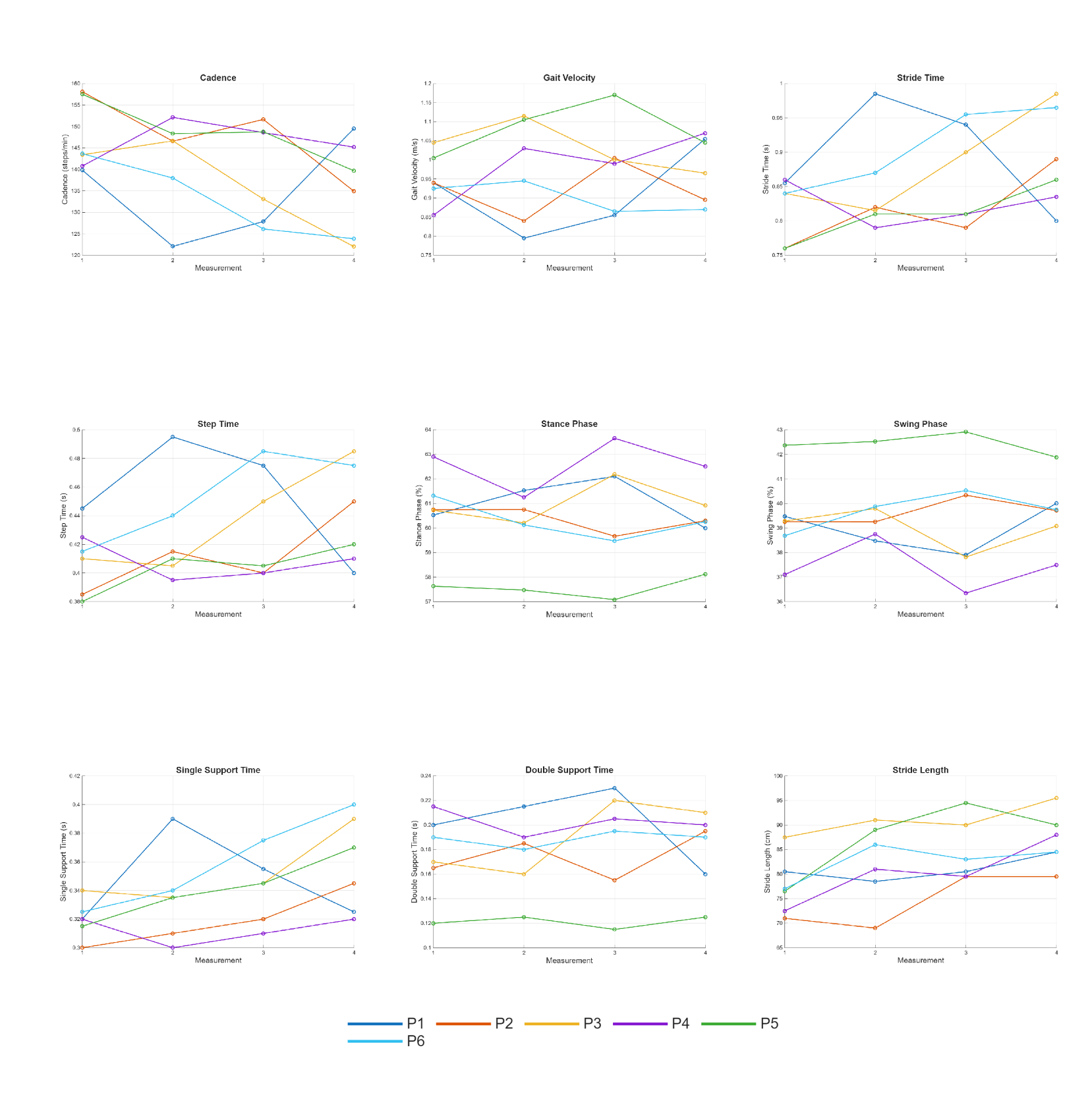


Figure SF 2 Spatio temporal parameters for all children with ACH for all four time points to display the individual development of each child. ACH = Achondroplasia.


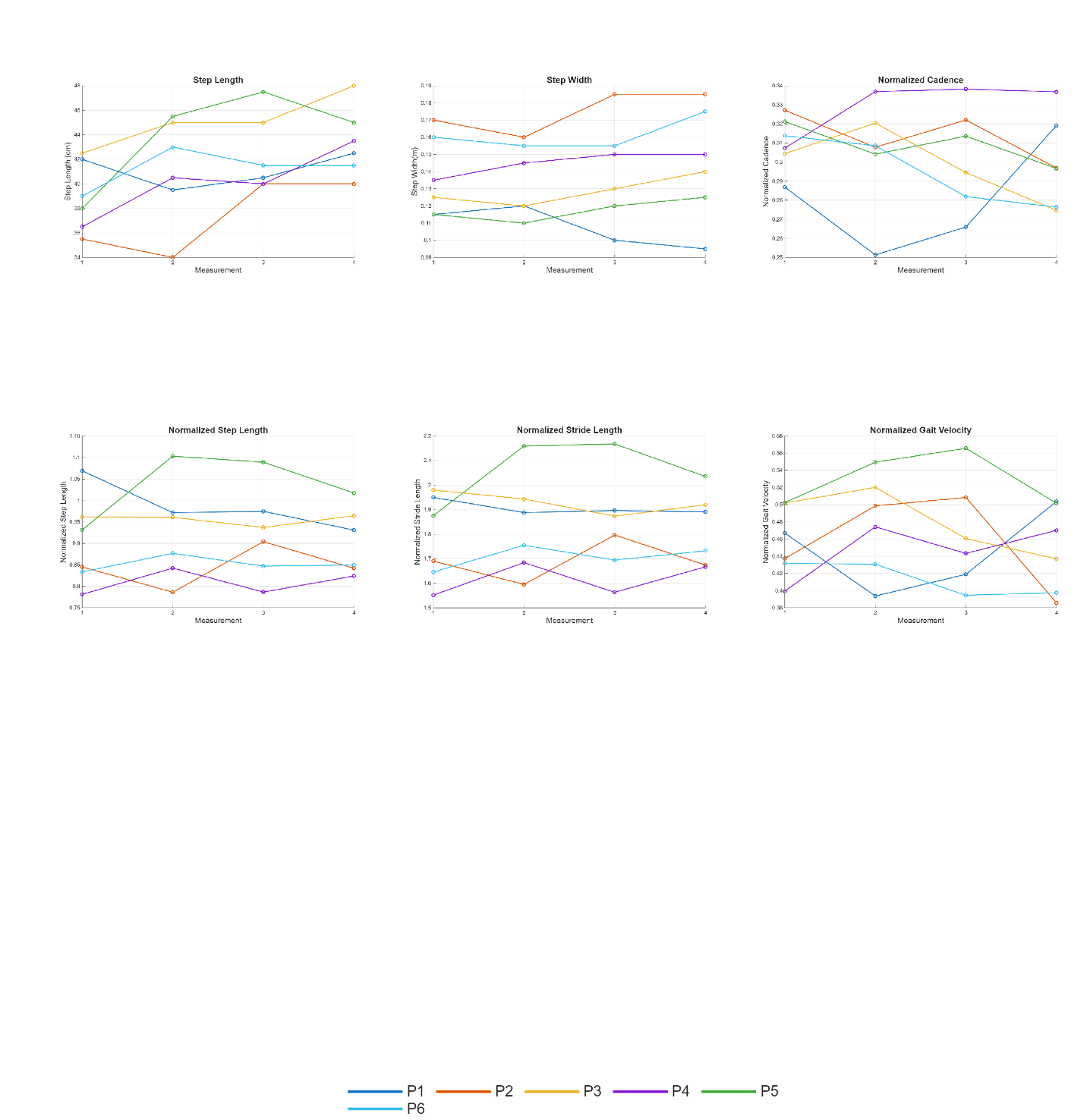


Figure SF 3 Second part of spatio temporal parameters for all children with ACH for all four time points to display the individual development of each child. ACH = Achondroplasia.


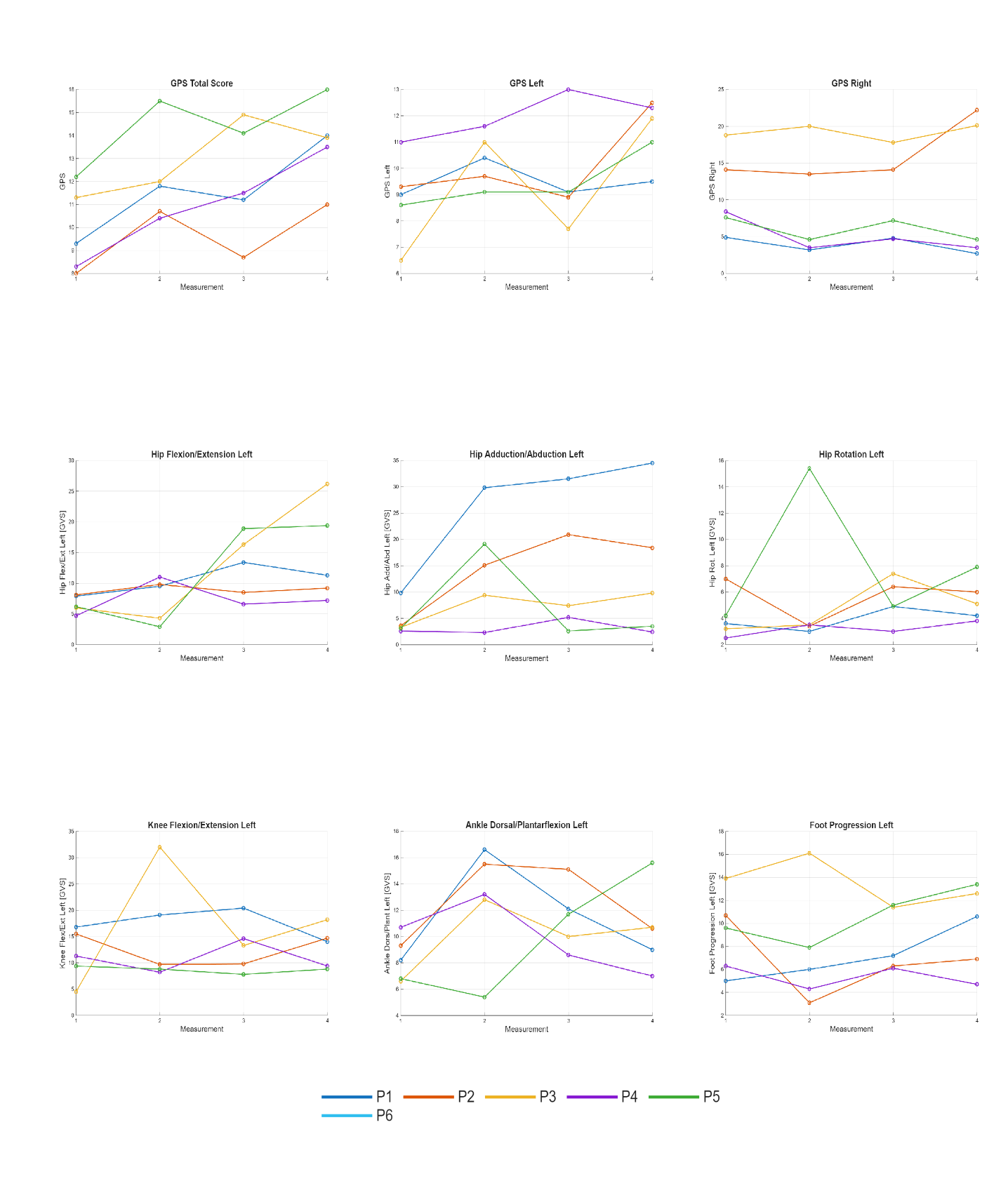


FigureSF 4 GPS and GVS parameters of the left side for all children with ACH for all four time points. GPS = Gait Profile Score, GVS = Gait Variable Score, ACH = Achondroplasia.


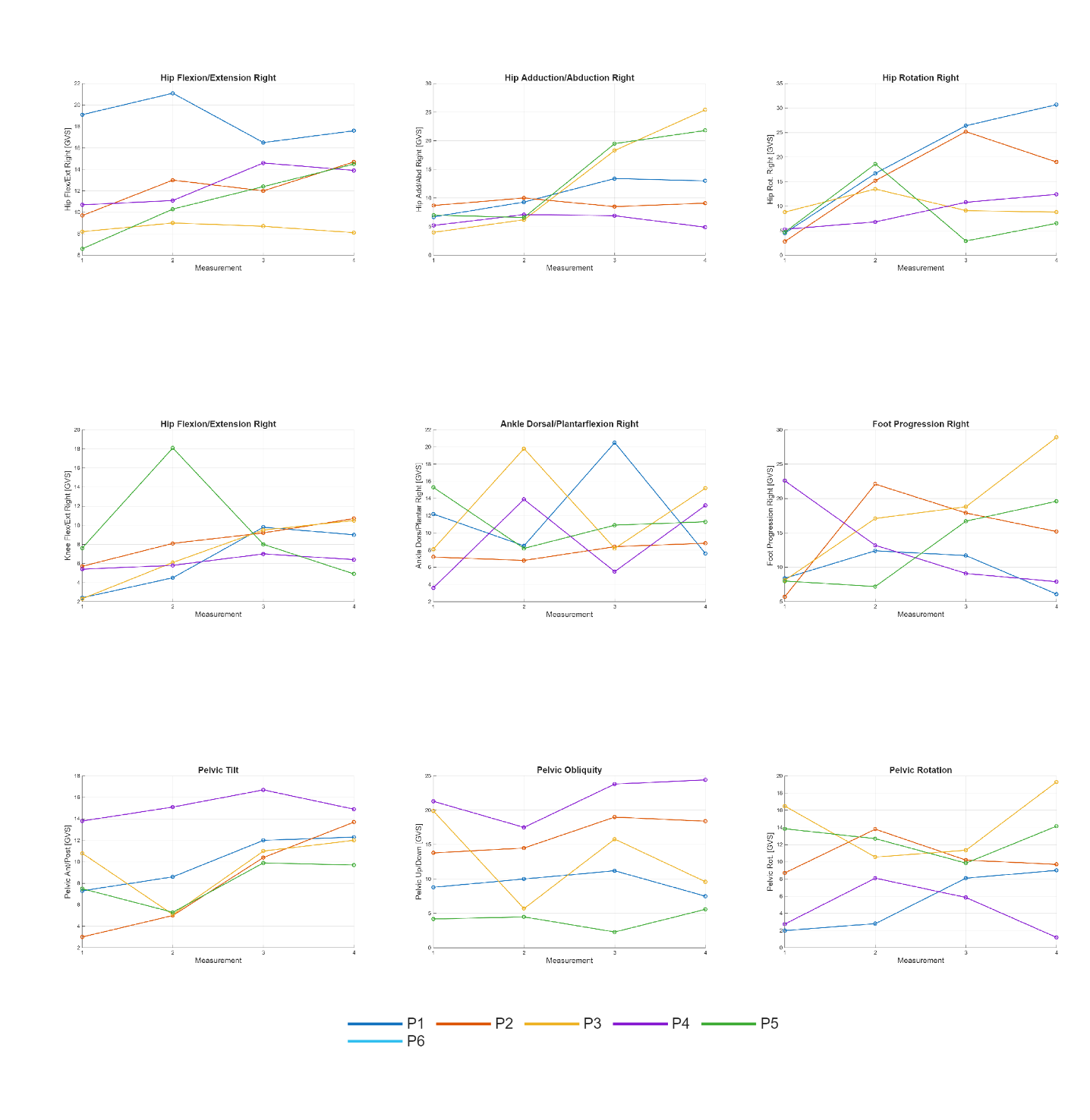


FigureSF 5 GPS and GVS parameters of the right side and the GVS pelvic parameters for all children with ACH for all four time points. GPS = Gait Profile Score, GVS = Gait Variable Score, ACH = Achondroplasia.


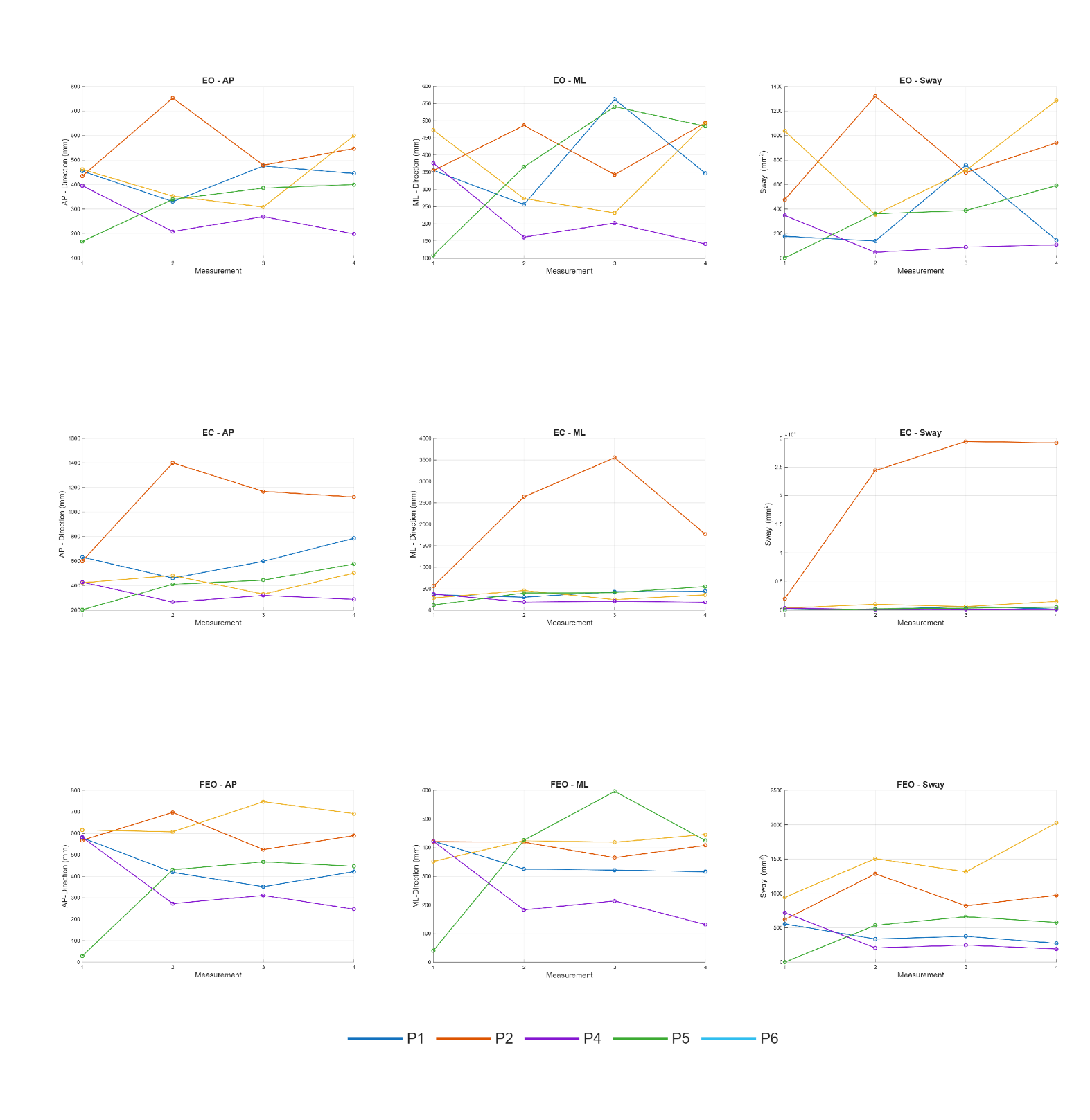


FigureSF 6 CoP and sway parameters for all children with ACH for all four time points. CoP = Center of pressure. ACH = Achondroplasia. EO = Eyes Open, EC = Eyes Closed, FEO = Foampad eyes open, AP = Anterior posterior, ML = Medio lateral.


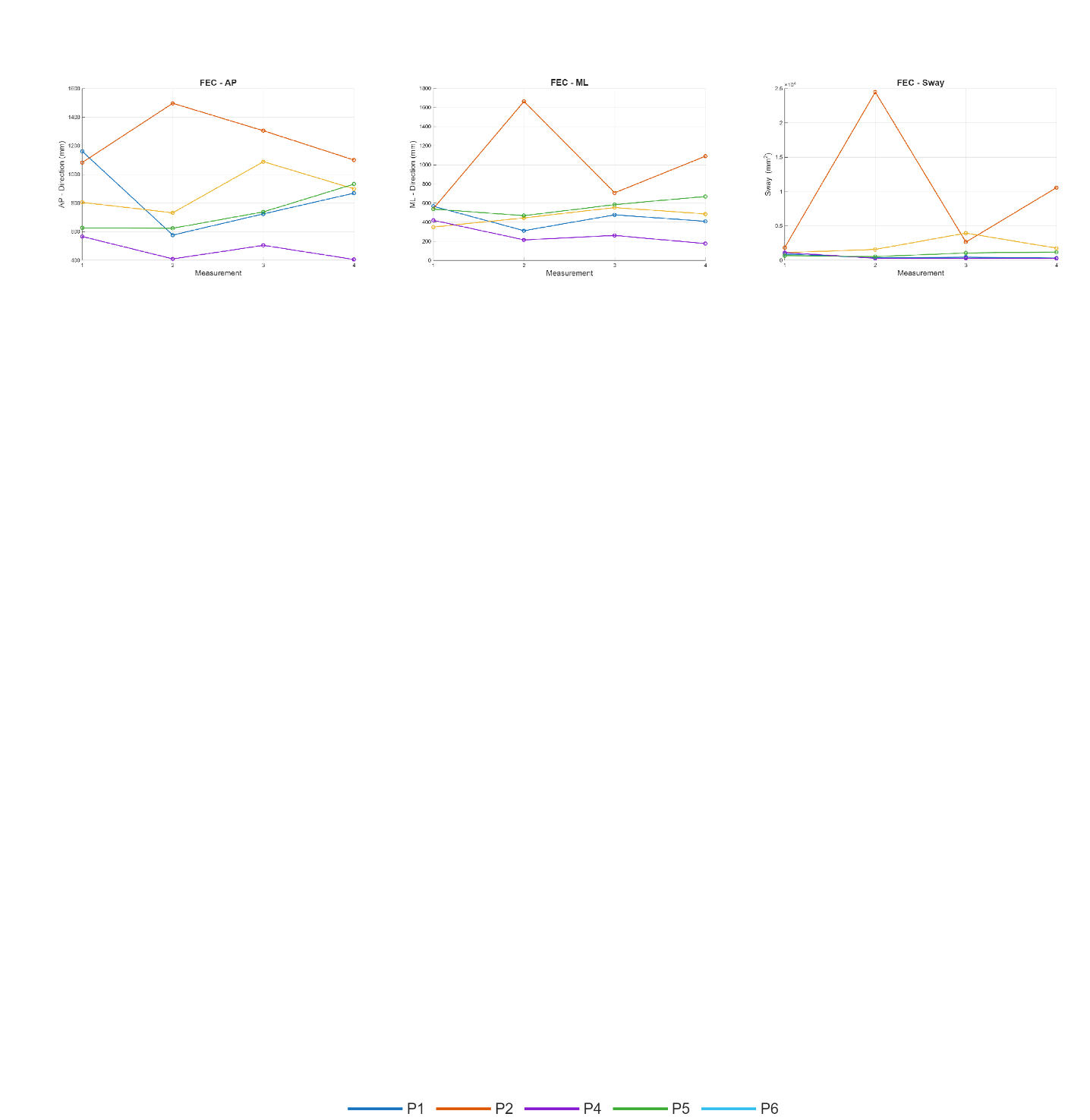


Figure SF 7 CoP and sway parameters for all children with ACH for all four time points. CoP = Center of pressure. ACH = Achondroplasia. FEC = Foampad eyes closed, AP = Anterior posterior, ML = Medio lateral.


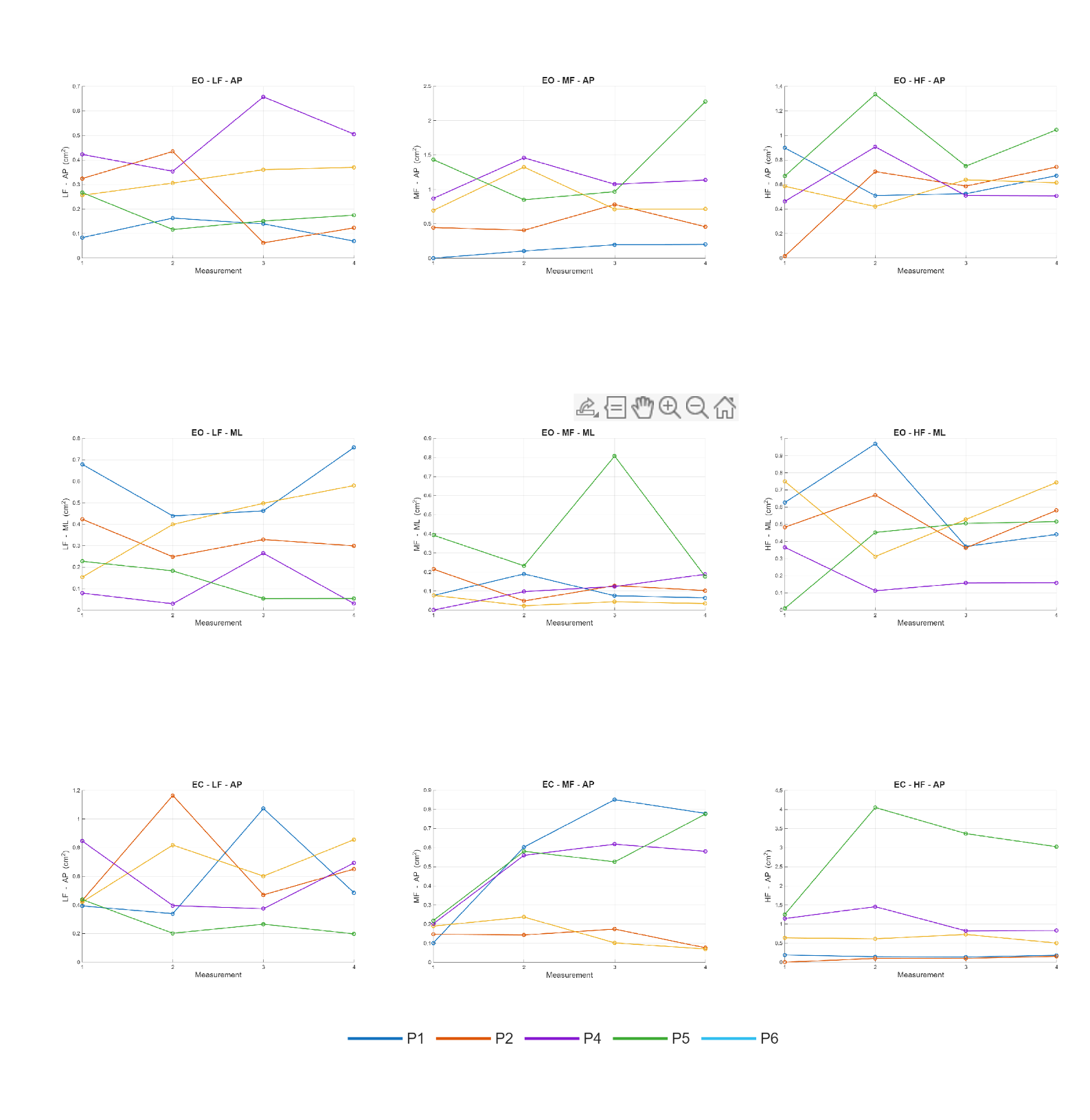


Figure SF 8 AUC parameters for the condition eyes open (EO) and closed (EC) for all children with ACH for all four time points. AUC = Area under the curve, ACH = Achondroplasia, LF = Low frequency, MF = Medium frequency, HF = High frequency, AP = Anterior posterior, ML = Medio lateral.


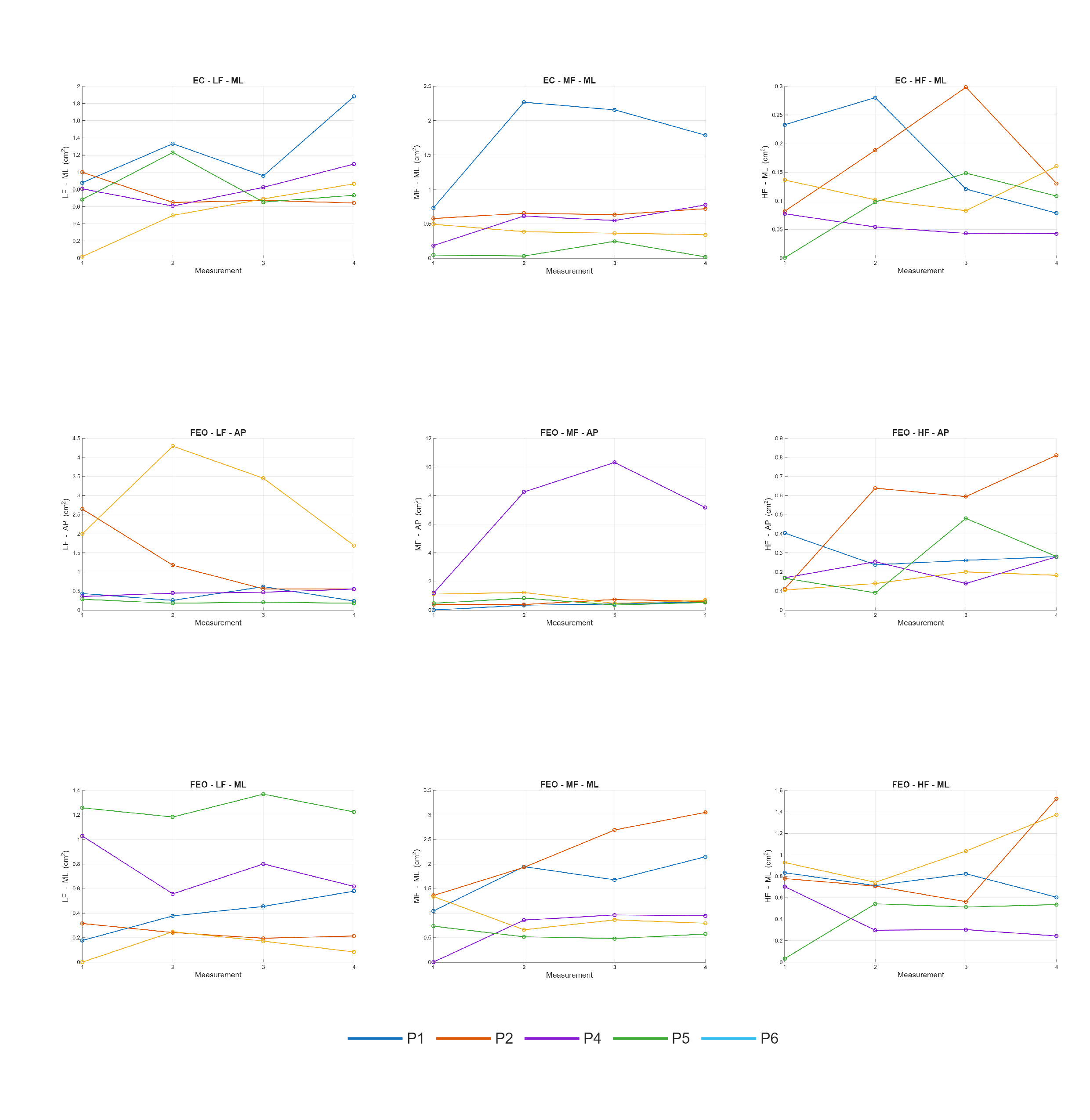


Figure SF 9 AUC parameters for the condition eyes closed (EC) and foampad eyes open (FEO) for all children with ACH for all four time points. AUC = Area under the curve, ACH = Achondroplasia, LF = Low frequency, MF = Medium frequency, HF = High frequency, AP = Anterior posterior, ML = Medio lateral.


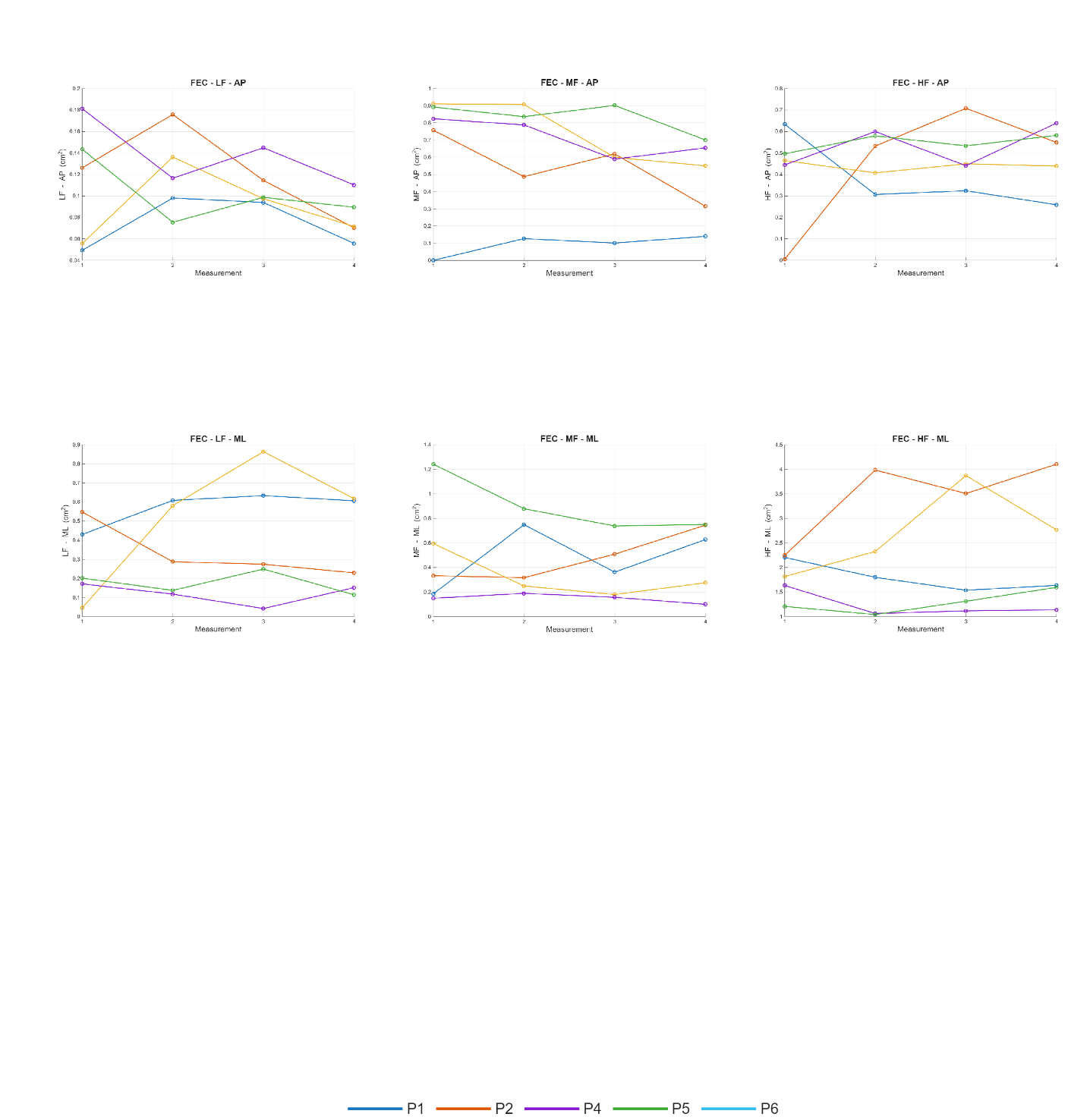


Figure SF 10 AUC parameters for the condition foampad eyes closed (FEC) for all children with ACH for all four time points. AUC = Area under the curve, ACH = Achondroplasia, LF = Low frequency, MF = Medium frequency, HF = High frequency, AP = Anterior posterior, ML = Medio lateral.

Table SF 1: Results of the npardLD F1-LD-F1 Model with ATS for the Factor Group for the anthropometric parameters. Post hoc effect size is presented as r. ATS = Anova Type Statistics, RTE = Relative Treatment effect, FL = Foot Leg. SiSta = Sitting to Standing. ACH = Achondroplasia. CAH = Children of average. T1 = Timepoint 1, T2 = Timepoint 2, T3 = Timepoint 3, T4 = Timepoint 4.

|  | **ANOVA Type Statistics (ATS)** | | |  |  | **T1** | | **T2** | | **T3** | | **T4** | |
| --- | --- | --- | --- | --- | --- | --- | --- | --- | --- | --- | --- | --- | --- |
| **Parameter** | **F** | **df** | **P** | **RTE ACH** | **RTE CAH** | **Post Hoc** | **Effect size** | **Post Hoc** | **Effect size** | **Post Hoc** | **Effect size** | **Post Hoc** | **Effect size** |
| Height | 68.73 | 1.00 | **0.00** | 0.12 | **0.59** | **0.00** | 0.66 | **0.00** | 0.66 | **0.00** | 0.65 | **0.00** | 0.67 |
| Weight | 0.01 | 1.00 | 0.91 | 0.49 | **0.50** | 1.00 | 0.04 | 1.00 | 0.01 | 1.00 | 0.06 | 1.00 | 0.01 |
| Age | 5.82 | 1.00 | **0.02** | **0.70** | 0.45 | 0.14 | 0.34 | 0.14 | 0.36 | 0.14 | 0.36 | 0.07 | 0.43 |
| Sitting Height | 0.77 | 1.00 | 0.38 | **0.59** | 0.48 | 0.86 | 0.05 | 0.86 | 0.19 | 0.86 | 0.18 | 0.74 | 0.24 |
| FL - Ratio | 136.80 | 1.00 | **0.00** | **0.90** | 0.40 | **0.00** | 0.68 | **0.00** | 0.68 | **0.00** | 0.68 | **0.00** | 0.68 |
| SiSta - Ratio | 27.22 | 1.00 | **0.00** | **0.90** | 0.40 | **0.00** | 0.68 | **0.00** | 0.68 | **0.00** | 0.68 | **0.00** | 0.68 |
| Pelvic Tilt | 27.22 | 1.00 | **0.00** | **0.81** | 0.42 | **0.03** | 0.39 | **0.00** | 0.61 | **0.01** | 0.53 | **0.00** | 0.60 |
| Leg Length | 98.85 | 1.00 | **0.00** | 0.10 | **0.60** | **0.00** | 0.68 | **0.00** | 0.68 | **0.00** | 0.68 | **0.00** | 0.68 |

Table SF 2: Results of the npardLD F1-LD-F1 Model with ATS for the Factor Time for the anthropometric parameters. Post hoc effect size is presented as r. ATS = Anova Type Statistics, FL = Foot Leg, SiSta = Sitting to Standing. ACH = Achondroplasia. CAH = Children of average. T1 = Timepoint 1, T2 = Timepoint 2, T3 = Timepoint 3, T4 = Timepoint 4.

|  |  |  |  | **ACH** | | **CAH** | | **ACH** | | **CAH** | | **ACH** | | **CAH** | |
| --- | --- | --- | --- | --- | --- | --- | --- | --- | --- | --- | --- | --- | --- | --- | --- |
|  | **ANOVA Type Statistics (ATS)** | | | **T1 - T2** | | **T1 - T2** | | **T2 - T3** | | **T2 - T3** | | **T3 - T4** | | **T3 - T4** | |
| **Parameter** | **F** | **df** | **P** | **Post Hoc** | **Effect size** | **Post Hoc** | **Effect size** | **Post Hoc** | **Effect size** | **Post Hoc** | **Effect size** | **Post Hoc** | **Effect size** | **Post Hoc** | **Effect size** |
| Height | 102.77 | 1.22 | **0.00** | 0.21 | 0.86 | **0.00** | 0.87 | 0.21 | 0.86 | **0.00** | 0.87 | 0.21 | 0.86 | **0.00** | 0.87 |
| Weight | 54.28 | 1.69 | **0.00** | 0.21 | 0.86 | **0.00** | 0.76 | 0.21 | 0.86 | **0.00** | 0.87 | 0.21 | 0.86 | **0.00** | 0.83 |
| Age | 177.33 | 1.64 | **0.00** | 0.21 | 0.86 | **0.00** | 0.87 | 0.21 | 0.86 | **0.00** | 0.87 | 0.21 | 0.86 | **0.00** | 0.87 |
| Sitting Height | 33.17 | 1.98 | **0.00** | 0.21 | 0.86 | **0.00** | 0.11 | 0.21 | 0.60 | **0.00** | 0.84 | 0.21 | 0.69 | **0.00** | 0.76 |
| FL - Ratio | 1.16 | 2.69 | 0.32 | 1.00 | 0.00 | 0.09 | 0.50 | 1.00 | 0.45 | 0.37 | 0.37 | 1.00 | 0.22 | 0.47 | 0.29 |
| SiSta - Ratio | 3.21 | 2.49 | **0.03** | 1.00 | 0.46 | **0.01** | 0.66 | 1.00 | 0.44 | 1.00 | 0.05 | 1.00 | 0.33 | 1.00 | 0.13 |
| Pelvic Tilt | 3.68 | 2.25 | **0.02** | 1.00 | 0.17 | 0.50 | 0.35 | 1.00 | 0.09 | 1.00 | 0.10 | 0.22 | 0.86 | 0.50 | 0.33 |
| Leg Length | 134.75 | 2.12 | **0.00** | 0.22 | 0.86 | **0.00** | 0.83 | 0.22 | 0.86 | **0.00** | 0.87 | 0.22 | 0.86 | **0.00** | 0.87 |

Table SF 3: Results of the npardLD F1-LD-F1 Model with ATS for the Factor Group for the spatio temporal parameters. Post hoc effect size is presented as r. ATS = Anova Type Statistics,RTE = Relative treatment effect, ACH = Achondroplasia. CAH = Children. T1 = Timepoint 1, T2 = Timepoint 2, T3 = Timepoint 3, T4 = Timepoint 4.

|  | **ANOVA Type Statistics (ATS)** | | |  |  | **T1** | | **T2** | | **T3** | | **T4** | |
| --- | --- | --- | --- | --- | --- | --- | --- | --- | --- | --- | --- | --- | --- |
| **Parameter** | **F** | **df** | **P** | **RTE ACH** | **RTE CAH** | **Post Hoc** | **Effect size** | **Post Hoc** | **Effect size** | **Post Hoc** | **Effect size** | **Post Hoc** | **Effect size** |
| Cadence | 14.23 | 1.00 | **0.00** | **0.73** | 0.44 | **0.02** | 0.51 | 0.08 | 0.40 | 0.10 | 0.35 | 0.35 | 0.10 |
| Gait Velocity | 99.18 | 1.00 | **0.00** | 0.13 | **0.59** | **0.00** | 0.65 | **0.00** | 0.63 | **0.00** | 0.60 | **0.00** | 0.64 |
| Stride Time | 15.17 | 1.00 | **0.00** | 0.27 | **0.56** | **0.03** | 0.50 | 0.08 | 0.40 | 0.08 | 0.35 | 0.08 | 0.37 |
| Step Time | 13.08 | 1.00 | **0.00** | 0.28 | **0.56** | **0.02** | 0.51 | 0.17 | 0.35 | 0.17 | 0.33 | 0.17 | 0.34 |
| Stance Phase | 8.04 | 1.00 | **0.01** | **0.78** | 0.43 | **0.02** | 0.51 | 0.06 | 0.40 | 0.06 | 0.40 | **0.01** | 0.56 |
| Swing Phase | 8.04 | 1.00 | **0.01** | 0.22 | **0.57** | **0.02** | 0.51 | 0.06 | 0.51 | 0.06 | 0.40 | **0.01** | 0.56 |
| Single Support Time | 37.37 | 1.00 | **0.00** | 0.20 | **0.58** | **0.00** | 0.65 | **0.01** | 0.49 | **0.00** | 0.59 | **0.01** | 0.50 |
| Double Support Time | 2.18 | 1.00 | 0.14 | 0.66 | 0.46 | 0.47 | 0.23 | 0.47 | 0.29 | 0.47 | 0.26 | 0.47 | 0.27 |
| Stride Length | 122.60 | 1.00 | **0.00** | 0.10 | **0.60** | **0.00** | 0.68 | **0.00** | 0.66 | **0.00** | 0.68 | **0.00** | 0.68 |
| Step Length | 124.97 | 1.00 | **0.00** | 0.10 | **0.60** | **0.00** | 0.68 | **0.00** | 0.66 | **0.00** | 0.68 | **0.00** | 0.68 |
| Step Width | 3.90 | 1.00 | **0.05** | **0.68** | 0.46 | 0.17 | 0.37 | 0.26 | 0.20 | 0.23 | 0.30 | 0.23 | 0.32 |
| Nor. Cadence | 73.31 | 1.00 | **0.00** | 0.15 | **0.59** | **0.00** | 0.61 | **0.00** | 0.63 | **0.00** | 0.57 | **0.00** | 0.54 |
| Nor. Step Length | 5.14 | 1.00 | **0.02** | **0.71** | 0.45 | 0.16 | 0.29 | 0.16 | 0.34 | 0.16 | 0.37 | 0.16 | 0.36 |
| Nor. Stride Length | 6.44 | 1.00 | **0.01** | **0.72** | 0.44 | 0.11 | 0.29 | 0.09 | 0.39 | 0.09 | 0.36 | 0.06 | 0.45 |
| Nor. Gait Velocity | 1.16 | 1.00 | 0.28 | 0.41 | 0.52 | 0.74 | 0.24 | 1.00 | 0.01 | 1.00 | 0.16 | 1.00 | 0.18 |

Table SF 4: Results of the npardLD F1-LD-F1 Model with ATS for the Factor Time for the spatio temporal parameters. Post hoc effect size is presented as r. ATS = Anova Type Statistics, ACH = Achondroplasia. CAH = Children,T1 = Timepoint 1, T2 = Timepoint 2, T3 = Timepoint 3, T4 = Timepoint 4.

|  |  |  |  | **ACH** | | **CAH** | | **ACH** | | **CAH** | | **ACH** | | **CAH** | |
| --- | --- | --- | --- | --- | --- | --- | --- | --- | --- | --- | --- | --- | --- | --- | --- |
|  | **ANOVA Type Statistics (ATS)** | | | **T1 - T2** | | **T1 - T2** | | **T2 - T3** | | **T2 - T3** | | **T3 - T4** | | **T3 - T4** | |
| **Parameter** | **F** | **df** | **P** | **Post Hoc** | **Effect size** | **Post Hoc** | **Effect size** | **Post Hoc** | **Effect size** | **Post Hoc** | **Effect size** | **Post Hoc** | **Effect size** | **Post Hoc** | **Effect size** |
| Cadence | 4.05 | 1.85 | **0.02** | 1.00 | 0.43 | 0.74 | 0.07 | 1.00 | 0.17 | 0.05 | 0.51 | 1.00 | 0.34 | 0.25 | 0.35 |
| Gait Velocity | 0.28 | 2.80 | 0.82 | 1.00 | 0.17 | 1.00 | 0.08 | 1.00 | 0.00 | 1.00 | 0.02 | 1.00 | 0.00 | 1.00 | 0.01 |
| Stride Time | 3.70 | 1.88 | 0.03 | 1.00 | 0.34 | 0.82 | 0.05 | 1.00 | 0.22 | 0.07 | 0.48 | 1.00 | 0.34 | 0.19 | 0.38 |
| Step Time | 2.35 | 1.95 | 0.10 | 1.00 | 0.43 | 0.67 | 0.14 | 1.00 | 0.17 | 0.21 | 0.42 | 1.00 | 0.17 | 0.67 | 0.22 |
| Stance Phase | 0.49 | 2.59 | 0.66 | 1.00 | 0.43 | 0.21 | 0.43 | 1.00 | 0.17 | 1.00 | 0.17 | 1.00 | 0.34 | 1.00 | 0.22 |
| Swing Phase | 0.49 | 2.59 | 0.66 | 1.00 | 0.43 | 0.21 | 0.43 | 1.00 | 0.17 | 1.00 | 0.17 | 1.00 | 0.34 | 1.00 | 0.22 |
| Single Support Time | 8.76 | 1.78 | **0.00** | 1.00 | 0.39 | 0.66 | 0.09 | 1.00 | 0.34 | **0.03** | 0.54 | 1.00 | 0.43 | **0.03** | 0.51 |
| Double Support Time | 0.11 | 2.57 | 0.93 | 1.00 | 0.00 | 1.00 | 0.01 | 1.00 | 0.34 | 1.00 | 0.01 | 1.00 | 0.17 | 1.00 | 0.04 |
| Stride Length | 12.60 | 2.54 | **0.00** | 0.42 | 0.60 | 0.51 | 0.13 | 0.56 | 0.26 | **0.02** | 0.61 | 0.56 | 0.44 | 0.16 | 0.40 |
| Step Length | 10.40 | 2.61 | **0.00** | 0.55 | 0.56 | 0.52 | 0.13 | 0.72 | 0.33 | 0.19 | 0.38 | 0.72 | 0.37 | 0.19 | 0.37 |
| Step Width | 1.70 | 1.80 | 0.19 | 1.00 | 0.35 | 0.07 | 0.52 | 1.00 | 0.33 | 0.84 | 0.22 | 1.00 | 0.45 | 1.00 | 0.14 |
| Nor. Cadence | 1.25 | 2.40 | 0.29 | 1.00 | 0.26 | 1.00 | 0.17 | 1.00 | 0.00 | 1.00 | 0.14 | 1.00 | 0.34 | 1.00 | 0.19 |
| Nor. Step Length | 0.22 | 2.06 | 0.81 | 1.00 | 0.09 | 1.00 | 0.02 | 1.00 | 0.26 | 1.00 | 0.00 | 1.00 | 0.34 | 1.00 | 0.02 |
| Nor. Stride Length | 0.15 | 1.95 | 0.85 | 1.00 | 0.34 | 1.00 | 0.10 | 1.00 | 0.09 | 1.00 | 0.06 | 1.00 | 0.09 | 1.00 | 0.14 |
| Nor. Gait Velocity | 0.68 | 2.10 | 0.52 | 1.00 | 0.34 | 1.00 | 0.08 | 1.00 | 0.34 | 1.00 | 0.09 | 1.00 | 0.09 | 1.00 | 0.07 |

Table SF 5: Results of the npardLD F1-LD-F1 Model with ATS for the Factor Time for the GPS and GVS parameters. Post hoc effect size is presented as r. ATS = Anova Type Statistics,GPS = Gait profile score, GVS = Gait variable score, ACH = Achondroplasia. CAH = Children,T1 = Timepoint 1, T2 = Timepoint 2, T3 = Timepoint 3, T4 = Timepoint 4.

|  |  |  |  | **ACH** | | **ACH** | | **ACH** | |
| --- | --- | --- | --- | --- | --- | --- | --- | --- | --- |
|  | **ANOVA Type Statistics (ATS)** | | | **T1 - T2** | | **T2 - T3** | | **T3 - T4** | |
| **Parameter** | **F** | **df** | **P** | **Post Hoc** | **Effect size** | **Post Hoc** | **Effect size** | **Post Hoc** | **Effect size** |
| **Overall GPS** | 9.58 | 1.75 | **0.00** | 0.21 | 0.86 | 0.68 | 0.17 | 0.59 | 0.43 |
| **Left** |  |  |  |  |  |  |  |  |  |
| GPS | 3.36 | 1.77 | **0.04** | 0.21 | 0.86 | 1.00 | 0.00 | 0.92 | 0.30 |
| Hip Flex/Ext | 13.59 | 1.54 | **0.00** | 0.28 | 0.60 | 0.28 | 0.69 | 0.28 | 0.60 |
| Hip Abd/Add | 2.36 | 1.93 | 0.10 | 1.00 | 0.00 | 0.71 | 0.60 | 0.88 | 0.43 |
| Hip Rotation | 0.44 | 1.73 | 0.61 | 1.00 | 0.17 | 1.00 | 0.17 | 1.00 | 0.00 |
| Knee Flex/Ext | 0.64 | 1.81 | 0.51 | 0.85 | 0.60 | 1.00 | 0.17 | 1.00 | 0.00 |
| Ankle Dors/Plantar | 1.78 | 2.38 | 0.16 | 1.00 | 0.43 | 1.00 | 0.43 | 1.00 | 0.26 |
| Foot Progression | 1.35 | 2.05 | 0.26 | 1.00 | 0.26 | 1.00 | 0.17 | 1.00 | 0.43 |
| **Right** |  |  |  |  |  |  |  |  |  |
| GPS | 1.35 | 2.05 | 0.26 | 0.35 | 0.84 | 0.42 | 0.43 | 0.42 | 0.51 |
| Hip Flex/Ext | 14.23 | 1.88 | **0.00** | 0.30 | 0.77 | 0.30 | 0.69 | 0.68 | 0.17 |
| Hip Abd/Add | 13.86 | 1.34 | **0.00** | 0.22 | 0.69 | 0.22 | 0.86 | 0.40 | 0.34 |
| Hip Rotation | 0.51 | 2.06 | 0.60 | 1.00 | 0.34 | 1.00 | 0.09 | 1.00 | 0.09 |
| Knee Flex/Ext | 0.61 | 1.75 | 0.52 | 1.00 | 0.43 | 1.00 | 0.34 | 1.00 | 0.43 |
| Ankle Dors/Plantar | 4.79 | 1.51 | **0.02** | 0.80 | 0.34 | 0.22 | 0.86 | 0.83 | 0.09 |
| Foot Progression | 2.11 | 1.74 | 0.13 | 1.00 | 0.43 | 1.00 | 0.30 | 1.00 | 0.51 |
| **Pelvic** |  |  |  |  |  |  |  |  |  |
| Tilt | 1.63 | 2.16 | 0.19 | 1.00 | 0.34 | 1.00 | 0.00 | 0.71 | 0.60 |
| Obliquity | 0.68 | 1.60 | 0.48 | 1.00 | 0.43 | 1.00 | 0.26 | 1.00 | 0.43 |
| Rotation | 0.56 | 1.47 | 0.52 | 1.00 | 0.00 | 1.00 | 0.17 | 1.00 | 0.51 |

Table SF 6: Results of the npardLD F1-LD-F1 Model with ATS for the Factor Group for the CoP parameters. Post hoc effect size is presented as r. ATS = Anova Type Statistics,RTE = Relative treatment effect, ACH = Achondroplasia. CAH = Children,T1 = Timepoint 1, T2 = Timepoint 2, T3 = Timepoint 3, T4 = Timepoint 4. CoP = Center of pressure, AP = anterior – posterior, ML = medio lateral.

|  | **ANOVA Type Statistics (ATS)** | | |  |  | **T1** | | **T2** | | **T3** | | **T4** | |
| --- | --- | --- | --- | --- | --- | --- | --- | --- | --- | --- | --- | --- | --- |
| **Parameter** | **F** | **df** | **P** | **RTE ACH** | **RTE CAH** | **Post Hoc P** | **Effect size** | **Post Hoc P** | **Effect size** | **Post Hoc P** | **Effect size** | **Post Hoc P** | **Effect size** |
| **EO** |  |  |  |  |  |  |  |  |  |  |  |  |  |
| CoP - AP | 4.78 | 1.00 | **0.03** | **0.70** | 0.45 | 0.20 | 0.29 | 0.20 | 0.32 | 0.20 | 0.38 | 0.20 | 0.37 |
| CoP - ML | 2.41 | 1.00 | 0.12 | **0.63** | 0.47 | 0.82 | 0.18 | 0.82 | 0.17 | 0.82 | 0.22 | 0.64 | 0.28 |
| Sway | 0.31 | 1.00 | 0.58 | **0.56** | 0.49 | 1.00 | 0.05 | 1.00 | 0.07 | 1.00 | 0.22 | 1.00 | 0.17 |
| **EC** |  |  |  |  |  |  |  |  |  |  |  |  |  |
| CoP - AP | 2.86 | 1.00 | 0.09 | **0.67** | 0.46 | 0.60 | 0.11 | 0.55 | 0.22 | 0.54 | 0.26 | 0.08 | 0.46 |
| CoP - ML | 1.24 | 1.00 | 0.27 | **0.61** | 0.47 | 1.00 | 0.10 | 1.00 | 0.17 | 1.00 | 0.16 | 0.39 | 0.32 |
| Sway | 0.11 | 1.00 | 0.74 | **0.54** | 0.49 | 1.00 | 0.16 | 1.00 | 0.05 | 0.80 | 0.25 | 1.00 | 0.19 |
| **FEO** |  |  |  |  |  |  |  |  |  |  |  |  |  |
| CoP - AP | 0.02 | 1.00 | 0.88 | **0.52** | **0.50** | 1.00 | 0.06 | 1.00 | 0.04 | 1.00 | 0.08 | 1.00 | 0.12 |
| CoP - ML | 0.36 | 1.00 | 0.55 | **0.55** | 0.49 | 1.00 | 0.02 | 1.00 | 0.13 | 1.00 | 0.06 | 1.00 | 0.07 |
| Sway | 0.59 | 1.00 | 0.44 | 0.41 | **0.52** | 0.51 | 0.30 | 1.00 | 0.12 | 1.00 | 0.07 | 1.00 | 0.10 |
| **FEC** |  |  |  |  |  |  |  |  |  |  |  |  |  |
| CoP - AP | 0.12 | 1.00 | 0.73 | **0.54** | 0.49 | 0.87 | 0.10 | 0.87 | 0.16 | 0.75 | 0.23 | 0.45 | 0.31 |
| CoP - ML | 0.11 | 1.00 | 0.74 | **0.54** | 0.49 | 1.00 | 0.11 | 1.00 | 0.06 | 1.00 | 0.22 | 1.00 | 0.12 |
| Sway | 0.37 | 1.00 | 0.54 | 0.42 | **0.52** | 0.57 | 0.29 | 1.00 | 0.16 | 1.00 | 0.02 | 1.00 | 0.08 |

Table SF 7: Results of the npardLD F1-LD-F1 Model with ATS for the Factor Time for the CoP parameters. Post hoc effect size is presented as r. ATS = Anova Type Statistics,GPS = Gait profile score, GVS = Gait variable score, ACH = Achondroplasia. CAH = Children,T1 = Timepoint 1, T2 = Timepoint 2, T3 = Timepoint 3, T4 = Timepoint 4. CoP = Center of pressure, AP = anterior – posterior, ML = medio lateral.

|  |  |  |  | **ACH** | | **ACH** | | **ACH** | | **CAH** | | **CAH** | | **CAH** | |
| --- | --- | --- | --- | --- | --- | --- | --- | --- | --- | --- | --- | --- | --- | --- | --- |
|  | **ANOVA Type Statistics (ATS)** | | | **T1 - T2** | | **T2 - T3** | | **T3 - T4** | | **T1 - T2** | | **T2 - T3** | | **T3 - T4** | |
| **Parameter** | **F** | **df** | **P** | **Post hoc P** | **Effect size** | **Post hoc P** | **Effect size** | **Post hoc P** | **Effect size** | **Post hoc P** | **Effect size** | **Post hoc P** | **Effect size** | **Post hoc P** | **Effect size** |
| **EO** |  |  |  |  |  |  |  |  |  |  |  |  |  |  |  |
| CoP - AP | 0.43 | 1.56 | 0.60 | 1.00 | 0.00 | 1.00 | 0.24 | 1.00 | 0.12 | 0.33 | 0.41 | 1.00 | 0.24 | 1.00 | 0.67 |
| CoP - ML | 0.34 | 1.76 | 0.68 | 1.00 | 0.00 | 1.00 | 0.24 | 1.00 | 0.00 | 1.00 | 0.19 | 0.92 | 0.31 | 1.00 | 0.07 |
| Sway | 0.83 | 2.02 | 0.44 | 1.00 | 0.00 | 1.00 | 0.24 | 1.00 | 0.24 | 0.48 | 0.08 | 0.48 | 0.37 | 0.95 | 0.07 |
| **EC** |  |  |  |  |  |  |  |  |  |  |  |  |  |  |  |
| CoP - AP | 0.07 | 1.59 | 0.89 | 1.00 | 0.24 | 1.00 | 0.12 | 1.00 | 0.48 | 0.61 | 0.15 | 0.61 | 0.28 | 0.61 | 0.31 |
| CoP - ML | 0.19 | 1.69 | 0.79 | 1.00 | 0.36 | 1.00 | 0.36 | 1.00 | 0.00 | 1.00 | 0.05 | 1.00 | 0.06 | 0.77 | 0.31 |
| Sway | 0.13 | 2.21 | 0.89 | 1.00 | 0.48 | 1.00 | 0.48 | 1.00 | 0.00 | 0.51 | 0.33 | 0.62 | 0.22 | 0.62 | 0.14 |
| **FEO** |  |  |  |  |  |  |  |  |  |  |  |  |  |  |  |
| CoP - AP | 0.56 | 1.51 | 0.52 | 1.00 | 0.00 | 1.00 | 0.00 | 1.00 | 0.00 | 1.00 | 0.08 | 1.00 | 0.13 | 0.42 | 0.35 |
| CoP - ML | 0.07 | 1.21 | 0.84 | 1.00 | 0.00 | 1.00 | 0.00 | 1.00 | 0.00 | 1.00 | 0.01 | 1.00 | 0.04 | 1.00 | 0.09 |
| Sway | 0.27 | 1.56 | 0.71 | 1.00 | 0.48 | 1.00 | 0.12 | 1.00 | 0.12 | 0.49 | 0.35 | 1.00 | 0.09 | 1.00 | 0.15 |
| **FEC** |  |  |  |  |  |  |  |  |  |  |  |  |  |  |  |
| CoP - AP | 1.87 | 2.26 | 0.15 | 1.00 | 0.36 | 1.00 | 0.36 | 1.00 | 0.12 | 0.39 | 0.28 | 0.05 | 0.53 | 0.50 | 0.15 |
| CoP - ML | 0.95 | 1.71 | 0.37 | 1.00 | 0.00 | 1.00 | 0.24 | 1.00 | 0.12 | 0.42 | 0.35 | 0.42 | 0.32 | 0.42 | 0.33 |
| Sway | 0.73 | 1.84 | 0.47 | 1.00 | 0.00 | 1.00 | 0.24 | 1.00 | 0.00 | 0.45 | 0.34 | 0.94 | 0.22 | 0.94 | 0.08 |

Table SF 8: Results of the npardLD F1-LD-F1 Model with ATS for the Factor Group for the AUC parameters. Post hoc effect size is presented as r. ATS = Anova Type Statistics, RTE = Relative treatment effect, ACH = Achondroplasia. CAH = Children of average height, ,T1 = Timepoint 1, T2 = Timepoint 2, T3 = Timepoint 3, T4 = Timepoint 4, LF = low frequency, MF = medium frequency, HF = high frequency, AP = anterior – posterior, ML = medio lateral.

|  | **ANOVA Type Statistics (ATS)** | | |  |  | **T1** | | **T2** | | **T3** | | **T4** | |
| --- | --- | --- | --- | --- | --- | --- | --- | --- | --- | --- | --- | --- | --- |
| **Parameter** | **F** | **df** | **P** | **RTE ACH** | **RTE CAH** | **Post Hoc** | **Effect size** | **Post Hoc** | **Effect size** | **Post Hoc** | **Effect size** | **Post Hoc** | **Effect size** |
| **EO** |  |  |  |  |  |  |  |  |  |  |  |  |  |
| LF-AP | 0.07 | 1.00 | 0.79 | **0.53** | 0.49 | 1.00 | 0.03 | 1.00 | 0.01 | 1.00 | 0.13 | 1.00 | 0.14 |
| MF-AP | 0.57 | 1.00 | 0.45 | **0.58** | 0.48 | 1.00 | 0.04 | 1.00 | 0.13 | 1.00 | 0.20 | 1.00 | 0.21 |
| HF-AP | 1.18 | 1.00 | 0.28 | **0.61** | 0.47 | 1.00 | 0.10 | 1.00 | 0.09 | 0.74 | 0.22 | 0.34 | 0.33 |
| LF-ML | 0.03 | 1.00 | 0.86 | **0.51** | **0.50** | 1.00 | 0.09 | 1.00 | 0.09 | 1.00 | 0.11 | 1.00 | 0.02 |
| MF-ML | 0.22 | 1.00 | 0.64 | **0.55** | 0.49 | 1.00 | 0.04 | 1.00 | 0.02 | 1.00 | 0.17 | 1.00 | 0.09 |
| HF-ML | 1.08 | 1.00 | 0.30 | **0.59** | 0.48 | 1.00 | 0.08 | 1.00 | 0.13 | 0.99 | 0.22 | 0.99 | 0.22 |
| **EC** |  |  |  |  |  |  |  |  |  |  |  |  |  |
| LF-AP | 6.33 | 1.00 | **0.01** | 0.25 | **0.56** | **0.00** | 0.65 | 0.11 | 0.40 | 0.25 | 0.29 | 0.25 | 0.27 |
| MF-AP | 0.24 | 1.00 | 0.63 | 0.56 | 0.49 | 1.00 | 0.05 | 1.00 | 0.04 | 1.00 | 0.20 | 1.00 | 0.19 |
| HF-AP | 1.10 | 1.00 | 0.30 | **0.61** | 0.47 | 1.00 | 0.01 | 1.00 | 0.11 | 0.43 | 0.28 | 0.39 | 0.32 |
| LF-ML | 0.03 | 1.00 | 0.87 | **0.51** | **0.50** | 1.00 | 0.19 | 1.00 | 0.02 | 0.64 | 0.27 | 1.00 | 0.07 |
| MF-ML | 0.02 | 1.00 | 0.89 | **0.52** | **0.50** | 1.00 | 0.14 | 1.00 | 0.13 | 0.99 | 0.22 | 1.00 | 0.10 |
| HF-ML | 0.41 | 1.00 | 0.52 | **0.57** | 0.48 | 1.00 | 0.01 | 1.00 | 0.15 | 1.00 | 0.10 | 1.00 | 0.19 |
| **FEO** |  |  |  |  |  |  |  |  |  |  |  |  |  |
| LF-AP | 80.02 | 1.00 | **0.00** | 0.12 | **0.59** | **0.00** | 0.65 | **0.00** | 0.65 | **0.00** | 0.61 | **0.01** | 0.51 |
| MF-AP | 0.37 | 1.00 | 0.55 | 0.42 | **0.52** | 0.64 | 0.27 | 1.00 | 0.19 | 1.00 | 0.10 | 1.00 | 0.66 |
| HF-AP | 0.23 | 1.00 | 0.63 | 0.45 | **0.51** | 1.00 | 0.42 | 0.74 | 0.22 | 0.45 | 0.31 | 1.00 | 0.11 |
| LF-ML | 4.84 | 1.00 | **0.03** | 0.36 | **0.53** | 0.30 | 0.34 | 1.00 | 0.11 | 1.00 | 0.08 | 0.48 | 0.27 |
| MF-ML | 0.08 | 1.00 | 0.78 | 0.47 | **0.51** | 1.00 | 0.02 | 1.00 | 0.05 | 1.00 | 0.66 | 1.00 | 0.14 |
| HF-ML | 0.03 | 1.00 | 0.86 | **0.51** | **0.50** | 1.00 | 0.02 | 1.00 | 0.13 | 1.00 | 0.05 | 1.00 | 0.05 |
| **FEC** |  |  |  |  |  |  |  |  |  |  |  |  |  |
| LF-AP | 0.27 | 1.00 | 0.60 | 0.44 | **0.51** | 1.00 | 0.17 | 1.00 | 0.78 | 1.00 | 0.14 | 1.00 | 0.01 |
| MF-AP | 0.14 | 1.00 | 0.71 | 0.45 | **0.51** | 0.89 | 0.23 | 0.91 | 0.20 | 1.00 | 0.02 | 1.00 | 0.02 |
| HF-AP | 0.05 | 1.00 | 0.82 | **0.52** | 0.49 | 1.00 | 0.15 | 1.00 | 0.17 | 1.00 | 0.17 | 0.45 | 0.31 |
| LF-ML | 1.83 | 1.00 | 0.18 | 0.38 | **0.53** | 0.82 | 0.21 | 0.50 | 0.29 | 1.00 | 0.08 | 1.00 | 0.13 |
| MF-ML | 0.36 | 1.00 | 0.55 | 0.43 | **0.51** | 1.00 | 0.13 | 0.99 | 0.22 | 1.00 | 0.09 | 1.00 | 0.19 |
| HF-ML | 0.15 | 1.00 | 0.70 | **0.55** | 0.49 | 1.00 | 0.17 | 1.00 | 0.07 | 0.89 | 0.23 | 1.00 | 0.16 |

Table SF 9: Results of the npardLD F1-LD-F1 Model with ATS for the Factor Group for the AUC parameters. Post hoc effect size is presented as r. ATS = Anova Type Statistics, RTE = Relative treatment effect, ACH = Achondroplasia. CAH = Children of average height, T1 = Timepoint 1, T2 = Timepoint 2, T3 = Timepoint 3, T4 = Timepoint 4, LF = low frequency, MF = medium frequency, HF = high frequency, AP = anterior – posterior, ML = medio lateral.

|  |  |  |  | **ACH** | | **CAH** | | **ACH** | | **CAH** | | **ACH** | | **CAH** | |
| --- | --- | --- | --- | --- | --- | --- | --- | --- | --- | --- | --- | --- | --- | --- | --- |
|  | **ANOVA Type Statistics (ATS)** | | | **T1 - T2** | | **T1 - T2** | | **T2 -T3** | | **T2 - T3** | | **T3 - T4** | | **T3 - T4** | |
| **Parameter** | **F** | **df** | **P** | **Post Hoc** | **Effect size** | **Post Hoc** | **Effect size** | **Post Hoc** | **Effect size** | **Post Hoc** | **Effect size** | **Post Hoc** | **Effect size** | **Post Hoc** | **Effect size** |
| **EO** |  |  |  |  |  |  |  |  |  |  |  |  |  |  |  |
| LF-AP | 0.59 | 2.02 | 0.55 | 1.00 | 0.00 | 0.63 | 0.35 | 0.63 | 0.72 | 1.00 | 0.15 | 1.00 | 0.12 | 1.00 | 0.16 |
| MF-AP | 0.44 | 1.96 | 0.64 | 1.00 | 0.12 | 0.86 | 0.31 | 1.00 | 0.00 | 1.00 | 0.04 | 1.00 | 0.36 | 1.00 | 0.13 |
| HF-AP | 1.26 | 1.99 | 0.28 | 1.00 | 0.12 | 1.00 | 0.10 | 1.00 | 0.24 | 1.00 | 0.15 | 1.00 | 0.48 | 1.00 | 0.02 |
| LF-ML | 1.08 | 2.29 | 0.35 | 1.00 | 0.00 | 0.19 | 0.46 | 1.00 | 0.36 | 0.28 | 0.41 | 1.00 | 0.36 | 1.00 | 0.18 |
| MF-ML | 0.78 | 2.21 | 0.47 | 1.00 | 0.00 | 0.06 | 0.55 | 1.00 | 0.36 | 0.62 | 0.27 | 1.00 | 0.24 | 0.91 | 0.16 |
| HF-ML | 0.98 | 1.66 | 0.36 | 1.00 | 0.00 | 1.00 | 0.02 | 1.00 | 0.36 | 0.41 | 0.39 | 1.00 | 0.00 | 1.00 | 0.01 |
| **EC** |  |  |  |  |  |  |  |  |  |  |  |  |  |  |  |
| LF-AP | 1.67 | 1.61 | 0.19 | 1.00 | 0.48 | 1.00 | 0.06 | 1.00 | 0.12 | 0.72 | 0.33 | 1.00 | 0.24 | 1.00 | 0.14 |
| MF-AP | 0.07 | 2.05 | 0.94 | 1.00 | 0.48 | 1.00 | 0.12 | 1.00 | 0.00 | 0.46 | 0.28 | 0.46 | 0.31 | 0.72 | 0.08 |
| HF-AP | 0.63 | 1.64 | 0.50 | 1.00 | 0.24 | 1.00 | 0.06 | 1.00 | 0.24 | 0.72 | 0.33 | 1.00 | 0.12 | 1.00 | 0.14 |
| LF-ML | 0.64 | 2.21 | 0.54 | 1.00 | 0.12 | 1.00 | 0.04 | 1.00 | 0.48 | 1.00 | 0.26 | 1.00 | 0.48 | 1.00 | 0.10 |
| MF-ML | 0.44 | 1.65 | 0.60 | 1.00 | 0.24 | 0.22 | 0.28 | 1.00 | 0.24 | 0.22 | 0.34 | 1.00 | 0.24 | 0.13 | 0.43 |
| HF-ML | 0.77 | 2.04 | 0.47 | 1.00 | 0.48 | 1.00 | 0.10 | 1.00 | 0.12 | 1.00 | 0.12 | 1.00 | 0.00 | 1.00 | 0.05 |
| **FOAMEO** |  |  |  |  |  |  |  |  |  |  |  |  |  |  |  |
| LF-AP | 0.29 | 2.18 | 0.77 | 1.00 | 0.24 | 1.00 | 0.13 | 1.00 | 0.36 | 1.00 | 0.17 | 1.00 | 0.12 | 1.00 | 0.14 |
| MF-AP | 0.07 | 1.27 | 0.85 | 1.00 | 0.36 | 0.82 | 0.24 | 1.00 | 0.36 | 1.00 | 0.01 | 1.00 | 0.12 | 0.82 | 0.27 |
| HF-AP | 1.36 | 2.05 | 0.26 | 1.00 | 0.24 | 1.00 | 0.13 | 1.00 | 0.12 | 1.00 | 0.08 | 1.00 | 0.60 | 1.00 | 0.14 |
| LF-ML | 0.49 | 1.91 | 0.60 | 1.00 | 0.24 | 1.00 | 0.19 | 1.00 | 0.12 | 0.37 | 0.38 | 1.00 | 0.24 | 1.00 | 0.21 |
| MF-ML | 1.97 | 1.80 | 0.14 | 1.00 | 0.24 | 0.34 | 0.37 | 1.00 | 0.24 | 0.69 | 0.21 | 0.89 | 0.60 | 0.69 | 0.08 |
| HF-ML | 0.71 | 1.26 | 0.43 | 1.00 | 0.24 | 1.00 | 0.16 | 1.00 | 0.24 | 1.00 | 0.24 | 1.00 | 0.36 | 1.00 | 0.18 |
| **FOAMEC** |  |  |  |  |  |  |  |  |  |  |  |  |  |  |  |
| LF-AP | 0.13 | 1.94 | 0.88 | 1.00 | 0.00 | 1.00 | 0.26 | 1.00 | 0.36 | 1.00 | 0.04 | 0.63 | 0.72 | 1.00 | 0.04 |
| MF-AP | 0.71 | 1.77 | 0.47 | 1.00 | 0.00 | 0.81 | 0.14 | 1.00 | 0.12 | 0.34 | 0.37 | 1.00 | 0.24 | 0.81 | 0.24 |
| HF-AP | 0.41 | 2.25 | 0.69 | 1.00 | 0.00 | 0.52 | 0.27 | 1.00 | 0.24 | 0.52 | 0.29 | 1.00 | 0.36 | 0.54 | 0.13 |
| LF-ML | 0.53 | 1.98 | 0.59 | 1.00 | 0.00 | 1.00 | 0.14 | 1.00 | 0.12 | 1.00 | 0.15 | 1.00 | 0.00 | 1.00 | 0.08 |
| MF-ML | 2.11 | 2.40 | 0.11 | 1.00 | 0.24 | 0.51 | 0.24 | 1.00 | 0.12 | 0.13 | 0.46 | 1.00 | 0.00 | 0.49 | 0.30 |
| HF-ML | 0.19 | 1.49 | 0.76 | 1.00 | 0.24 | 0.55 | 0.36 | 1.00 | 0.24 | 1.00 | 0.09 | 1.00 | 0.00 | 1.00 | 0.13 |

Table SF 10: SRD thresholds and surpassings for each child with achondroplasia at each time point for the spatio temporal parameters. SRD = Smalles real difference, ACH = Achondroplasia

|  | **Measurement Time 1 - 2** | | | | | | |
| --- | --- | --- | --- | --- | --- | --- | --- |
| **Parameter** | **SRD** | **P1** | **P2** | **P3** | **P4** | **P5** | **P6** |
| Cadence (steps/min) | 13.05 | **-17.75** | -11.54 | 3.23 | 11.35 | -9.19 | -5.69 |
| Gait Velocity (m/s) | 0.24 | **-0.15** | -0.10 | 0.07 | **0.18** | 0.10 | 0.02 |
| Stride Time (s) | 0.07 | **0.13** | 0.06 | -0.03 | -0.07 | 0.05 | 0.03 |
| Step Time (s) | 0.06 | **0.05** | 0.03 | 0.00 | -0.03 | 0.03 | 0.03 |
| Stance Phase (%) | 1.69 | 1.00 | 0.00 | -0.52 | **-1.66** | -0.15 | -1.19 |
| Swing Phase (%) | 1.69 | -1.00 | 0.00 | 0.52 | **1.66** | 0.15 | 1.19 |
| Single Support Time (s) | 0.04 | **0.07** | 0.01 | -0.01 | -0.02 | 0.02 | 0.02 |
| Double Support Time (s) | 0.03 | 0.02 | 0.02 | -0.01 | -0.03 | 0.01 | -0.01 |
| Stride Length (cm) | 12.02 | -2.00 | -2.00 | 3.50 | 8.50 | 12.50 | 9.00 |
| Step Length (cm) | 8.15 | -2.50 | -1.50 | 2.50 | 4.00 | **7.50** | 4.00 |
| Step Width (m) | 0.02 | 0.01 | -0.01 | -0.01 | 0.01 | 0.00 | -0.01 |
| Nor. Cadence | 0.04 | **-0.04** | -0.02 | 0.02 | 0.03 | -0.02 | -0.01 |
| Nor. Step Length | 0.25 | -0.10 | -0.06 | 0.00 | 0.06 | 0.17 | 0.04 |
| Nor. Stride Length | 0.46 | -0.06 | -0.10 | -0.04 | 0.13 | 0.28 | 0.11 |
| Nor. Gait Velocity | 0.12 | -0.07 | 0.06 | 0.02 | 0.07 | 0.05 | 0.00 |
|  | **Measurement Time 2 - 3** | | | | | | |
| **Parameter** | **SRD** | **P1** | **P2** | **P3** | **P4** | **P5** | **P6** |
| Cadence (steps/min) | 18.05 | 5.78 | 5.07 | -13.57 | -3.54 | 0.43 | -11.87 |
| Gait Velocity (m/s) | 0.27 | 0.06 | 0.17 | -0.12 | -0.04 | 0.06 | -0.08 |
| Stride Time (s) | 0.10 | -0.05 | -0.03 | **0.09** | 0.02 | 0.00 | **0.09** |
| Step Time (s) | 0.05 | -0.02 | -0.02 | **0.05** | 0.01 | -0.01 | **0.05** |
| Stance Phase (%) | 2.10 | 0.57 | -1.09 | 1.98 | 2.41 | -0.39 | -0.65 |
| Swing Phase (%) | 2.10 | -0.57 | 1.09 | -1.98 | -2.41 | 0.39 | 0.65 |
| Single Support Time (s) | 0.02 | **-0.04** | 0.01 | 0.01 | 0.01 | 0.01 | **0.04** |
| Double Support Time (s) | 0.05 | 0.02 | -0.03 | **0.06** | 0.02 | -0.01 | 0.02 |
| Stride Length (cm) | 6.15 | 2.00 | **10.50** | -1.00 | -1.50 | 5.50 | -3.00 |
| Step Length (cm) | 2.45 | 1.00 | **6.00** | 0.00 | -0.50 | 2.00 | -1.50 |
| Step Width (m) | 0.02 | **-0.02** | **0.03** | 0.01 | 0.00 | 0.01 | 0.00 |
| Nor. Cadence | 0.04 | 0.01 | 0.01 | -0.03 | 0.00 | 0.01 | -0.03 |
| Nor. Step Length | 0.18 | 0.00 | 0.12 | -0.02 | -0.06 | -0.01 | -0.03 |
| Nor. Stride Length | 0.23 | 0.01 | 0.20 | -0.07 | -0.12 | 0.01 | -0.06 |
| Nor. Gait Velocity | 0.09 | 0.03 | 0.01 | -0.06 | -0.03 | 0.02 | -0.04 |
|  | **Measurement Time 3 - 4** | | | | | | |
| **Parameter** | **SRD** | **P1** | **P2** | **P3** | **P4** | **P5** | **P6** |
| Cadence (steps/min) | 38.19 | 21.68 | -16.75 | -11.06 | -3.40 | -9.07 | -2.27 |
| Gait Velocity (m/s) | 0.29 | 0.20 | -0.11 | -0.04 | 0.08 | -0.13 | 0.01 |
| Stride Time (s) | 0.22 | -0.14 | 0.10 | 0.09 | 0.02 | 0.05 | 0.01 |
| Step Time (s) | 0.11 | **-0.08** | 0.05 | 0.04 | 0.01 | 0.02 | -0.01 |
| Stance Phase (%) | 1.55 | -2.11 | 0.63 | -1.27 | -1.15 | 1.04 | 0.78 |
| Swing Phase (%) | 1.55 | 2.11 | -0.63 | 1.27 | 1.15 | -1.04 | -0.78 |
| Single Support Time (s) | 0.06 | -0.03 | 0.03 | 0.05 | 0.01 | 0.03 | 0.03 |
| Double Support Time (s) | 0.06 | -0.07 | 0.04 | -0.01 | -0.01 | 0.01 | -0.01 |
| Stride Length (cm) | 5.84 | 4.00 | 0.00 | 5.50 | 8.50 | -4.50 | 1.50 |
| Step Length (cm) | 3.40 | 2.00 | 0.00 | 3.00 | 3.50 | -2.50 | 0.00 |
| Step Width (m) | 0.02 | -0.01 | 0.00 | 0.01 | 0.00 | 0.01 | **0.02** |
| Nor. Cadence | 0.08 | 0.05 | -0.03 | -0.02 | 0.00 | -0.02 | -0.01 |
| Nor. Step Length | 0.13 | -0.04 | -0.06 | 0.03 | 0.04 | -0.07 | 0.00 |
| Nor. Stride Length | 0.27 | -0.01 | -0.12 | 0.05 | 0.10 | -0.13 | 0.04 |
| Nor. Gait Velocity | 0.21 | 0.08 | -0.12 | -0.02 | 0.03 | -0.06 | 0.00 |

Table SF 11: SRD thresholds and surpassings for each child with achondroplasia at each time point for the GPS and GVS parameters. SRD = Smalles real difference, ACH = Achondroplasia. GPS = Gait profile score, GVS = Gait variable score.

|  | **Measurement Time 1 - 2** | | | | | | |
| --- | --- | --- | --- | --- | --- | --- | --- |
| **Parameter** | **SRD** | **P1** | **P2** | **P3** | **P4** | **P5** | **P6** |
| **GPS** | 0.51 | **2.50** | **0.70** | **2.10** | **3.30** | **2.10** | **1.40** |
| **Left** |  |  |  |  |  |  |  |
| GPS | 1.54 | 0.40 | 0.60 | 0.50 | **4.00** | **1.60** | **1.70** |
| Hip Ext/ Flexion | 13.57 | -1.70 | -3.30 | 7.10 | **20.00** | 11.50 | 6.10 |
| Hip Add/Abduction | 1.10 | -0.30 | 0.70 | -0.60 | **-3.60** | 0.30 | 1.00 |
| Hip Rotation | 20.98 | 11.20 | 2.30 | -5.80 | **27.50** | -3.10 | -0.60 |
| Knee Ext/Flexion | 4.57 | -0.10 | **6.20** | **6.20** | 2.50 | -1.40 | 2.80 |
| Ankle Dors/Plantarflexion | 6.52 | -3.00 | **-7.60** | 2.20 | -2.00 | -1.70 | 1.50 |
| Foot Progression | 5.11 | -1.70 | 1.20 | -4.90 | -3.00 | 1.40 | 2.00 |
| **Right** |  |  |  |  |  |  |  |
| GPS | 3.12 | **3.30** | 0.40 | **3.70** | 2.10 | 2.60 | 1.30 |
| Hip Ext/ Flexion | 7.81 | 2.20 | -0.40 | **10.70** | **12.20** | **12.40** | 4.70 |
| Hip Add/Abduction | 3.46 | 1.50 | -0.50 | 2.10 | 2.40 | **3.80** | 0.40 |
| Hip Rotation | 13.17 | 10.50 | -3.70 | -0.40 | 11.70 | 10.30 | -7.10 |
| Knee Ext/Flexion | 15.32 | 9.50 | **16.40** | 9.00 | -9.40 | -0.80 | 1.90 |
| Ankle Dors/Plantarflexion | 8.41 | 1.30 | -5.70 | 1.30 | -2.20 | -6.30 | 1.20 |
| Foot Progression | 0.00 | **0.70** | **-3.80** | **0.30** | **3.40** | **0.80** | **5.10** |
| **Pelvic** |  |  |  |  |  |  |  |
| Tilt | 7.33 | -5.95 | -1.15 | 2.10 | 4.95 | 3.35 | 5.95 |
| Obliquity | 1.14 | -0.20 | -0.40 | 0.45 | 0.55 | 1.00 | 0.10 |
| Rotation | 0.00 | **6.60** | **-0.15** | **-1.00** | **-0.15** | **1.00** | **-0.80** |
|  | **Measurement Time 2 - 3** | | | | | | |
| **Parameter** | **SRD** | **P1** | **P2** | **P3** | **P4** | **P5** | **P6** |
| **GPS** | 3.29 | -0.60 | 2.90 | 1.10 | -1.40 | **4.40** | -1.30 |
| **Left** |  |  |  |  |  |  |  |
| GPS | 4.59 | -0.80 | 1.40 | 0.00 | -2.30 | 3.90 | -1.30 |
| Hip Ext/ Flexion | 11.94 | **12.00** | **16.00** | 4.40 | 1.70 | 5.80 | -2.00 |
| Hip Add/Abduction | 3.54 | 2.90 | -0.20 | 1.90 | 3.00 | **3.90** | -0.50 |
| Hip Rotation | 14.17 | -10.50 | 1.30 | 0.10 | **-18.70** | 6.40 | -1.00 |
| Knee Ext/Flexion | 8.02 | -6.00 | -0.40 | -2.80 | -4.60 | 6.30 | 2.60 |
| Ankle Dors/Plantarflexion | 4.23 | **5.30** | 3.20 | **-4.70** | 1.80 | 3.70 | 1.90 |
| Foot Progression | 13.78 | 1.60 | -2.20 | 1.20 | 2.60 | 5.70 | -4.60 |
| **Right** |  |  |  |  |  |  |  |
| GPS | 3.75 | -1.00 | 3.50 | 2.10 | 0.80 | **4.10** | -1.50 |
| Hip Ext/ Flexion | 5.50 | **12.10** | **12.90** | 4.30 | **9.70** | **10.00** | -4.40 |
| Hip Add/Abduction | 3.26 | **4.00** | 1.40 | **5.30** | 1.10 | **3.40** | 1.20 |
| Hip Rotation | 13.14 | -10.10 | 12.00 | 1.60 | -11.60 | -8.40 | 2.70 |
| Knee Ext/Flexion | 14.12 | -11.60 | -4.20 | 1.70 | -4.10 | 9.50 | -3.10 |
| Ankle Dors/Plantarflexion | 3.84 | 3.40 | **5.90** | 1.60 | **4.60** | **8.20** | 1.20 |
| Foot Progression | 9.66 | 4.50 | 6.30 | -2.20 | -4.50 | 5.30 | -3.60 |
| **Pelvic** |  |  |  |  |  |  |  |
| Tilt | 4.59 | 0.80 | -2.85 | 2.45 | 3.20 | 1.35 | -3.60 |
| Obliquity | 1.08 | **-1.55** | 0.35 | 0.35 | 0.95 | -0.25 | **1.35** |
| Rotation | 4.28 | **-10.00** | -0.60 | 2.00 | -0.95 | 1.40 | -1.90 |
|  | **Measurement Time 3 - 4** | | | | | | |
| **Parameter** | **SRD** | **P1** | **P2** | **P3** | **P4** | **P5** | **P6** |
| **GPS** | 2.95 | 2.80 | -1.00 | 2.00 | 1.90 | -1.10 | 0.40 |
| **Left** |  |  |  |  |  |  |  |
| GPS | 3.90 | 3.60 | -0.70 | 1.90 | 1.50 | -2.10 | 0.70 |
| Hip Ext/ Flexion | 8.58 | **9.90** | 0.50 | 3.40 | 3.00 | -2.50 | 2.40 |
| Hip Add/Abduction | 3.40 | -2.80 | 0.20 | -0.70 | -0.40 | -2.30 | 0.80 |
| Hip Rotation | 8.82 | 3.00 | -6.40 | 4.90 | 4.90 | -5.20 | 1.00 |
| Knee Ext/Flexion | 4.64 | 2.80 | -4.50 | 0.70 | -1.60 | 3.90 | -2.30 |
| Ankle Dors/Plantarflexion | 4.96 | -2.20 | 0.60 | 1.20 | -1.40 | 1.80 | -2.10 |
| Foot Progression | 11.40 | -2.10 | 2.30 | -1.20 | -2.60 | -8.10 | 1.10 |
| **Right** |  |  |  |  |  |  |  |
| GPS | 3.05 | 2.70 | -0.70 | 2.10 | 2.40 | -0.40 | 0.60 |
| Hip Ext/ Flexion | 10.33 | 7.10 | 2.30 | -0.80 | 4.30 | -6.20 | -0.30 |
| Hip Add/Abduction | 2.70 | 1.60 | -0.10 | -0.80 | 1.50 | 1.00 | -0.60 |
| Hip Rotation | 9.79 | -3.10 | **-12.90** | 0.40 | 7.00 | 7.70 | 0.40 |
| Knee Ext/Flexion | 4.00 | 3.10 | -2.70 | **10.10** | -1.20 | 2.90 | 0.80 |
| Ankle Dors/Plantarflexion | 3.68 | 0.30 | 1.00 | -1.80 | -0.20 | 0.30 | **-3.70** |
| Foot Progression | 0.00 | **-0.60** | **0.60** | **3.30** | **3.50** | **0.90** | **-0.50** |
| **Pelvic** |  |  |  |  |  |  |  |
| Tilt | 7.16 | **7.95** | 4.30 | 4.10 | 3.00 | -3.55 | 3.15 |
| Obliquity | 0.24 | **0.25** | 0.15 | **0.50** | **1.35** | **-0.95** | 0.10 |
| Rotation | 2.36 | 1.80 | 0.40 | -1.00 | -0.10 | **4.05** | 2.05 |

Table SF 12: SRD thresholds and surpassings for each child with achondroplasia at each point for the CoP parameters. SRD = Smalles real difference, ACH = Achondroplasia. CoP = Center of pressure, AP = anterior – posterior, ML = medio lateral.

| **Measurement Time 1 - 2** | | | | | | |
| --- | --- | --- | --- | --- | --- | --- |
| **Parameter** | **SRD** | **P1** | **P2** | **P4** | **P5** | **P6** |
| **EO** |  |  |  |  |  |  |
| CoP – AP (mm) | 69.84 | **-124.65** | **318.50** | **-109.48** | **-187.20** | **171.77** |
| CoP – ML (mm) | 292.86 | -99.42 | 131.09 | -199.36 | -215.02 | 257.04 |
| Sway (mm^2^) | 751.96 | -38.33 | **843.69** | -681.53 | -300.61 | 360.80 |
| **EC** |  |  |  |  |  |  |
| CoP – AP (mm) | 379.99 | -170.76 | **801.51** | 57.97 | -162.59 | 208.44 |
| CoP – ML (mm) | 342.78 | -59.39 | **2080.10** | 176.24 | -182.27 | 281.12 |
| Sway (mm^2^) | 171.61 | -150.43 | **22395.69** | **663.08** | **-199.84** | **225.29** |
| **FEO** |  |  |  |  |  |  |
| CoP – AP (mm) | 381.59 | -161.43 | 129.89 | -8.58 | -308.80 | **401.87** |
| CoP – ML (mm) | 25.84 | **-97.61** | -1.71 | **72.42** | **-239.44** | **385.45** |
| Sway (mm^2^) | 740.24 | -220.25 | 666.38 | 564.63 | -513.57 | 537.10 |
| **FEC** |  |  |  |  |  |  |
| CoP – AP (mm) | 551.32 | **-584.90** | 414.04 | -73.01 | -156.69 | -2.52 |
| CoP – ML (mm) | 299.30 | -255.87 | **1122.38** | 95.70 | -204.60 | 69.98 |
| Sway (mm^2^) | 774.70 | -562.80 | **22638.92** | 461.49 | **-837.26** | -134.18 |
| **Measurement Time 2 - 3** | | | | | | |
| **Parameter** | **SRD** | **P1** | **P2** | **P4** | **P5** | **P6** |
| **EO** |  |  |  |  |  |  |
| CoP – AP (mm) | 133.99 | **145.23** | **-274.13** | -44.31 | 60.74 | 45.77 |
| CoP – ML (mm) | 400.31 | 305.69 | -142.80 | -41.60 | 40.93 | 175.18 |
| Sway (mm^2^) | 536.61 | **617.84** | **-621.57** | 359.92 | 42.13 | 25.16 |
| **EC** |  |  |  |  |  |  |
| CoP – AP (mm) | 197.46 | 136.78 | **-233.24** | -151.18 | 53.42 | 33.33 |
| CoP – ML (mm) | 341.40 | 128.26 | **911.38** | -213.47 | 18.58 | 9.95 |
| Sway (mm^2^) | 406.94 | **477.87** | **5088.20** | -399.41 | 26.41 | 128.86 |
| **FEO** |  |  |  |  |  |  |
| CoP – AP (mm) | 177.23 | -66.24 | -173.21 | 140.23 | 38.26 | 36.40 |
| CoP – ML (mm) | 0.00 | **-4.10** | **-54.01** | **-5.31** | **31.12** | **170.49** |
| Sway (mm^2^) | 0.00 | **38.68** | **-465.33** | **-192.80** | **41.10** | **124.88** |
| **FEC** |  |  |  |  |  |  |
| CoP – AP (mm) | 0.00 | **147.82** | **-191.89** | **357.18** | **94.70** | **114.64** |
| CoP – ML (mm) | 0.00 | **165.72** | **-955.65** | **108.52** | **45.55** | **116.27** |
| Sway (mm^2^) | 648.54 | 103.65 | **-21826.78** | **2371.82** | 8.32 | 536.33 |
| **Measurement Time 3 - 4** | | | | | | |
| **Parameter** | **SRD** | **P1** | **P2** | **P4** | **P5** | **P6** |
| **EO** |  |  |  |  |  |  |
| CoP – AP (mm) | 306.06 | -30.89 | 68.19 | 291.14 | -70.58 | 14.48 |
| CoP – ML (mm) | 295.06 | -215.26 | 151.01 | 258.90 | -60.98 | -57.13 |
| Sway (mm^2^) | 804.26 | -611.22 | 243.05 | 570.71 | 18.94 | 204.32 |
| **EC** |  |  |  |  |  |  |
| CoP – AP (mm) | 0.00 | **187.86** | **-44.86** | **171.42** | **-31.52** | **132.06** |
| CoP – ML (mm) | 177.08 | 12.29 | **-1781.54** | 109.22 | -22.50 | 140.43 |
| Sway (mm^2^) | 684.64 | -435.32 | **-9243.96** | **914.87** | 19.26 | 163.55 |
| **FEO** |  |  |  |  |  |  |
| CoP – AP (mm) | 0.00 | **69.81** | **65.00** | **-55.87** | **-64.38** | **-20.56** |
| CoP – ML (mm) | 59.66 | -5.29 | 42.78 | 27.13 | **-82.50** | **-171.63** |
| Sway (mm^2^) | 0.00 | **-100.28** | **153.90** | **713.21** | **-56.30** | **-82.86** |
| **FEC** |  |  |  |  |  |  |
| CoP – AP (mm) | 173.55 | 145.07 | **-204.65** | **-189.91** | -98.95 | **193.91** |
| CoP – ML (mm) | 0.00 | **-67.17** | **383.17** | **-66.96** | **-84.40** | **85.02** |
| Sway (mm^2^) | 1066.02 | -124.43 | **7929.93** | **-2185.15** | -20.32 | 139.15 |

Table SF 13: SRD thresholds and surpassings for each child with achondroplasia at each point for the AUC parameters. SRD = Smalles real difference, ACH = Achondroplasia. AUC =Area under the curve,LF = low frequency, MF = medium frequency, HF = high frequency, AP = anterior – posterior, ML = medio lateral.

| **Measurement Time 1 - 2** | | | | | | |
| --- | --- | --- | --- | --- | --- | --- |
| **Parameter** | **SRD** | **P1** | **P2** | **P4** | **P5** | **P6** |
| **EO** |  |  |  |  |  |  |
| LF-AP | 0.25 | 0.08 | 0.05 | -0.07 | -0.15 | 0.10 |
| MF-AP | 1.06 | -0.04 | 0.59 | -0.58 | -0.39 | 0.69 |
| HF-AP | 0.10 | **-0.17** | **0.67** | **-0.24** | **-0.17** | **0.25** |
| LF-ML | 0.07 | -0.05 | **0.11** | **-0.17** | -0.05 | **0.10** |
| MF-ML | 0.42 | -0.16 | 0.19 | **-0.44** | -0.25 | **0.44** |
| HF-ML | 0.52 | -0.05 | 0.40 | -0.45 | -0.24 | 0.50 |
| **EC** |  |  |  |  |  |  |
| LF-AP | 0.00 | **0.00** | **0.36** | **0.36** | **-0.05** | **0.10** |
| MF-AP | 0.25 | -0.03 | **2.80** | **0.45** | **-0.35** | **0.48** |
| HF-AP | 0.35 | -0.20 | **1.54** | 0.08 | -0.11 | **0.43** |
| LF-ML | 0.10 | -0.01 | **0.11** | -0.03 | -0.02 | **0.10** |
| MF-ML | 0.27 | -0.18 | **2.30** | 0.09 | -0.11 | **0.33** |
| HF-ML | 0.42 | -0.01 | **7.08** | 0.36 | -0.17 | **0.53** |
| **FEO** |  |  |  |  |  |  |
| LF-AP | 0.32 | 0.03 | -0.08 | 0.20 | -0.07 | 0.25 |
| MF-AP | 1.03 | -0.47 | 0.90 | 0.57 | -0.68 | 0.86 |
| HF-AP | 0.23 | -0.22 | -0.07 | -0.18 | **-0.41** | **0.51** |
| LF-ML | 0.17 | 0.05 | 0.08 | -0.06 | -0.07 | 0.13 |
| MF-ML | 0.39 | -0.27 | -0.04 | -0.06 | -0.33 | **0.53** |
| HF-ML | 0.17 | -0.06 | 0.08 | **0.18** | **-0.26** | **0.54** |
| **FEC** |  |  |  |  |  |  |
| LF-AP | 0.15 | -0.05 | **0.56** | -0.02 | **-0.35** | 0.04 |
| MF-AP | 0.37 | -0.36 | **1.74** | **0.51** | **-0.57** | -0.17 |
| HF-AP | 0.70 | **-0.75** | **1.21** | -0.38 | -0.04 | 0.11 |
| LF-ML | 0.06 | -0.01 | **0.14** | 0.01 | **-0.06** | -0.04 |
| MF-ML | 0.42 | -0.40 | **1.52** | -0.21 | **-0.46** | -0.12 |
| HF-ML | 0.80 | -0.29 | **5.36** | 0.42 | -0.12 | 0.05 |
| **Measurement Time 2 - 3** | | | | | | |
| **Parameter** | **SRD** | **P1** | **P2** | **P4** | **P5** | **P6** |
| **EO** |  |  |  |  |  |  |
| LF-AP (cm^2^) | 0.15 | -0.02 | 0.05 | **0.30** | 0.03 | 0.09 |
| MF-AP (cm^2^) | 0.44 | 0.38 | -0.38 | 0.12 | 0.02 | -0.12 |
| HF-AP (cm^2^) | 0.18 | **0.22** | **-0.59** | 0.02 | 0.08 | 0.10 |
| LF-ML (cm^2^) | 0.15 | **0.24** | -0.11 | 0.08 | 0.02 | 0.03 |
| MF-ML (cm^2^) | 0.58 | **0.58** | -0.31 | 0.22 | 0.05 | 0.05 |
| HF-ML (cm^2^) | 0.76 | 0.73 | -0.22 | -0.02 | 0.06 | 0.25 |
| **EC** |  |  |  |  |  |  |
| LF-AP (cm^2^) | 0.11 | 0.03 | 0.06 | -0.06 | -0.01 | 0.00 |
| MF-AP (cm^2^) | 0.23 | 0.12 | **-0.68** | **-0.37** | 0.02 | 0.19 |
| HF-AP (cm^2^) | 0.26 | 0.22 | -0.11 | -0.02 | -0.02 | -0.07 |
| LF-ML (cm^2^) | 0.24 | 0.21 | 0.11 | -0.02 | -0.01 | 0.05 |
| MF-ML (cm^2^) | 0.28 | **0.36** | **-0.84** | 0.02 | 0.03 | 0.09 |
| HF-ML (cm^2^) | 0.63 | 0.35 | **2.06** | -0.48 | 0.02 | -0.04 |
| **FEO** |  |  |  |  |  |  |
| LF-AP (cm^2^) | 0.32 | 0.06 | **0.39** | 0.08 | -0.05 | -0.08 |
| MF-AP (cm^2^) | 0.30 | 0.24 | -0.27 | **0.76** | 0.20 | 0.10 |
| HF-AP (cm^2^) | 0.00 | **-0.04** | **-0.15** | **0.29** | **0.01** | **-0.03** |
| LF-ML (cm^2^) | 0.04 | 0.00 | **-0.04** | 0.03 | 0.02 | -0.03 |
| MF-ML (cm^2^) | 0.35 | 0.13 | -0.20 | 0.07 | 0.02 | 0.18 |
| HF-ML (cm^2^) | 0.08 | 0.04 | -0.05 | 0.03 | -0.01 | **0.28** |
| **FEC** |  |  |  |  |  |  |
| LF-AP (cm^2^) | 0.13 | -0.08 | **-0.39** | **0.19** | -0.07 | -0.03 |
| MF-AP (cm^2^) | 0.70 | -0.14 | -0.48 | **1.54** | 0.05 | 0.27 |
| HF-AP (cm^2^) | 0.21 | 0.10 | **-1.04** | **0.34** | 0.20 | 0.07 |
| LF-ML (cm^2^) | 0.19 | 0.08 | -0.11 | -0.05 | 0.01 | **0.19** |
| MF-ML (cm^2^) | 0.30 | **0.33** | **-1.60** | **0.55** | -0.03 | 0.27 |
| HF-ML (cm^2^) | 0.40 | 0.24 | **-4.90** | 0.09 | 0.07 | 0.05 |
| **Measurement Time 3 - 4** | | | | | | |
| **Parameter** | **SRD** | **P1** | **P2** | **P4** | **P5** | **P6** |
| **EO** |  |  |  |  |  |  |
| LF-AP (cm^2^) | 0.00 | **-0.07** | **0.01** | **-0.15** | **0.02** | **0.01** |
| MF-AP (cm^2^) | 0.62 | -0.32 | 0.06 | **1.31** | 0.15 | 0.16 |
| HF-AP (cm^2^) | 0.32 | -0.02 | 0.30 | 0.30 | -0.03 | 0.08 |
| LF-ML (cm^2^) | 0.18 | **-0.23** | -0.01 | -0.03 | -0.01 | 0.06 |
| MF-ML (cm^2^) | 0.64 | -0.63 | 0.22 | 0.22 | 0.00 | 0.01 |
| HF-ML (cm^2^) | 0.71 | -0.59 | 0.25 | 0.32 | -0.07 | -0.07 |
| **EC** |  |  |  |  |  |  |
| LF-AP (cm^2^) | 0.23 | -0.10 | -0.04 | **0.25** | 0.05 | 0.05 |
| MF-AP (cm^2^) | 0.47 | -0.23 | -0.34 | **0.92** | -0.03 | 0.18 |
| HF-AP (cm^2^) | 0.33 | 0.27 | **-0.37** | 0.09 | -0.02 | 0.23 |
| LF-ML (cm^2^) | 0.31 | -0.23 | -0.17 | 0.08 | 0.00 | -0.04 |
| MF-ML (cm^2^) | 0.60 | -0.38 | **-1.76** | 0.08 | -0.03 | 0.15 |
| HF-ML (cm^2^) | 0.28 | -0.13 | **-3.15** | 0.18 | 0.02 | 0.22 |
| **FEO** |  |  |  |  |  |  |
| LF-AP (cm^2^) | 0.09 | -0.02 | **-0.20** | **0.12** | 0.02 | **-0.09** |
| MF-AP (cm^2^) | 0.00 | **-0.18** | **0.47** | **0.36** | **-0.07** | **-0.02** |
| HF-AP (cm^2^) | 0.35 | 0.09 | **0.96** | 0.34 | -0.06 | 0.02 |
| LF-ML (cm^2^) | 0.02 | **-0.04** | **-0.03** | **-0.03** | -0.01 | **0.04** |
| MF-ML (cm^2^) | 0.27 | **-0.30** | 0.07 | -0.20 | -0.07 | -0.16 |
| HF-ML (cm^2^) | 0.00 | **-0.01** | **0.05** | **-0.03** | **-0.04** | **-0.25** |
| **FEC** |  |  |  |  |  |  |
| LF-AP (cm^2^) | 0.20 | 0.11 | **0.27** | **0.24** | 0.10 | -0.06 |
| MF-AP (cm^2^) | 0.92 | 0.01 | 0.60 | **-1.10** | 0.02 | 0.28 |
| HF-AP (cm^2^) | 0.00 | **0.29** | **0.49** | **0.08** | **-0.34** | **0.30** |
| LF-ML (cm^2^) | 0.23 | -0.08 | **0.35** | 0.07 | 0.00 | -0.10 |
| MF-ML (cm^2^) | 0.61 | -0.40 | **1.85** | **-0.71** | 0.02 | -0.02 |
| HF-ML (cm^2^) | 0.33 | -0.07 | **1.05** | -0.16 | -0.12 | 0.14 |
